# Predicting heart failure onset in the general population using a novel data-mining artificial intelligence method

**DOI:** 10.1038/s41598-023-31600-0

**Published:** 2023-03-16

**Authors:** Yohei Miyashita, Tatsuro Hitsumoto, Hiroki Fukuda, Jiyoong Kim, Takashi Washio, Masafumi Kitakaze

**Affiliations:** 1 Department of Legal Medicine, Osaka University Graduate School of Medicine, 2-2 Yamadaoka, Suita, Osaka Japan; 2 Department of Clinical Research and Development, National Cerebral and Cardiovascular Center, 6-1 Kishibe-Shimmachi, Suita, Osaka Japan; 3 Kim Cardiovascular Clinic, 3-6-8 Katsuyama, Tennoji-ku, Osaka, Japan; 4 The Institute of Scientific and Industrial Research, Osaka University, 1-1 Yamadaoka, Suita, Osaka Japan; 5 Hanwa Memorial Hospital, 3-5-8 Minamisumiyoshi, Sumiyoshi-ku, Osaka, 558-0041 Japan; 6 The Osaka Medical Research Foundation for Intractable Diseases, 2-6-29 Abikohigashi, Sumiyoshi-ku, Osaka, Japan

**Keywords:** Computational biology and bioinformatics, Cardiology, Health care, Risk factors

## Abstract

We aimed to identify combinations of clinical factors that predict heart failure (HF) onset using a novel limitless-arity multiple-testing procedure (LAMP). We also determined if increases in numbers of predictive combinations of factors increases the probability of developing HF. We recruited people without HF who received health check-ups in 2010, who were followed annually for 4 years. Using 32,547 people, LAMP was performed to identify combinations of factors of fewer than four factors that could predict the onset of HF. The ability of the method to predict the probability of HF onset based on the number of matching predictive combinations of factors was determined in 275,658 people. We identified 549 combinations of factors for the onset of HF. Then we classified 275,658 people into six groups who had 0, 1–50, 51–100, 101–150, 151–200 or 201–250 predictive combinations of factors for the onset of HF. We found that the probability of HF progressively increased as the number of predictive combinations of factors increased. We identified combinations of variables that predict HF onset. An increased number of matching predictive combinations for the onset of HF increased the probability of HF onset.

## Introduction

Since the pathophysiology of heart failure (HF) is characterized by myocardial necrosis or apoptosis that causes irreversible cellular damage, establishing methods to prevent and treat HF is crucial. Hypertension (HT), type 2 diabetes mellitus (T2D), primary/secondary cardiomyopathy, valvular heart disease, and coronary artery diseases prime and contribute to HF^1^. Other factors such as renal dysfunction, anemia, age, and sex may also contribute to the onset of HF. However, each of these factors alone cannot accurately predict the onset of HF because the essence of HF pathophysiology is a multiplexity of many clinical, medical, physical, and habitual factors, unlike single factor-related diseases, such as genetic diseases. Therefore, a quantitative formula to predict the occurrence of HF and identify the high- or low-risk population is needed. Recently, machine learning has been progressed and applied to the area of the prediction of HF. There is an abundance of HF risk prediction models that had sufficient discriminative ability, although few are externally validated^2^. Wang et al.^3^ and Wu et al.^4^ showed the prediction of the onset of HF at 2 years and 0.5 years using machine learning such as random forest method and support vector machine, respectively. However, the variables that determine the onset of HF were not clarified and these studies are not validated using another cohort as an external validation. Furthermore, the time window for the prediction is relatively short. Several recent investigations^5–7^ revealed the artificial intelligence (AI)-based prediction of the onset of HF, however, either the variables to link to the onset of HF or the probabilities of the onset of HF have not been clarified. To conquer these unresolved issues, we have recently implemented novel advances in statistical testing that allowed us to analyze all significant combinations of clinical variables via the limitless-arity multiple-testing procedure (LAMP)^8,9^.

We hypothesized that the use of LAMP may clarify the significant combinations of clinical variables to predict the onset of HF, and that the individuals who have more combinations of factors for prediction of the onset of HF are likely to have the higher probability of the onset of HF. To establish such combinations of factors and their ability to predict the occurrence of HF, we first determined the predictive combinations of clinical, medical, physical, and habitual variables that are associated with the incidence of HF for 4 years among approximately 30,000 general Japanese people using LAMP. Second, we determined if increases in the number of matched combinations of factors increase the probability of HF occurrence in approximately 270,000 general people for 4 years.

## Methods

### Study design and participants

This was a retrospective observational study in Japan, and the informed consent for the retrospective study was waived with the Ethical Guidelines for Medical and Biological Research Involving Human Subjects issued by Ministry of Education, Culture, Sports, Science and Technology, Ministry of Health, Labour and Welfare, and Ministry of Economy, Trade and Industry in Japan. We analyzed healthcare insurance claims data obtained from the Japan Medical Data Center (JMDC) in Tokyo. The database contains standardized eligibility and claims data provided by health insurance societies for approximately 4.5 million insured individuals, including data from employees of general corporations and their family members. The database contains all medical treatments received by insured individuals at all treatment facilities and a comprehensive record of all treatments administered to a given patient. We removed the decoding indexes and analyzed the personal data with unlinkable anonymization.

The study protocol was approved by the ethics committee of the National Cerebral and Cardiovascular Center (M22-49, M24-51). On the basis of the Japanese Clinical Research Guidelines, the committee decided that patient informed consent was not essential for inclusion in this study because of the retrospective observational nature of the study. Instead, JMDC made a public announcement in accordance with the ethics committee’s request and the Japanese Clinical Research Guidelines. The study was performed following the principles of the Declaration of Helsinki and the Japanese Ethical Guidelines for Clinical Research.

### Protocols

In the database of the approximately 4.5 million people, we set the entry criteria of (1) the presence of the complete dataset of the consecutive period of 4 years following 2010, (2) no diagnosis of HF at the first year, (3) evidence of the presence or absence of diagnosis of HF during 4 years. Under these entry criteria, we narrowed down the database and obtained the complete dataset of 308,205 people. We then randomly allocated 32,547 people for the analysis cohort (Protocol 1) and remaining of 275,658 people for the validation cohort (Protocol 2) using the random numbers table. Because we have had several general experiences that about 30,000 people with 1% of incidence are enough to obtain the significant combinations of factors, and indeed we have experienced that we need several thousands people with about 10% of incidence to obtain the sufficient combinations of factors to identify the worthening of HF^10^, we selected about 30,000 people to find the meaningful and sufficient combinations of factors to detect the onset of HF for Protocol 1.

### Protocol 1

In 32,547 Japanese cohort, we obtained 288 clinical, medical, habitual, and physical variables at 2010, including sex; age; urinary sugar levels (borderline, 1 + , 2 + , 3 + , and 4 +); urinary protein levels (borderline, 1 + , 2 + , 3 + , and 4 +); plasma LDL and HDL cholesterol and triglyceride levels (mg/L); plasma HbA1c levels (%); body mass index; systolic and diastolic blood pressure (mmHg); plasma uric acid levels (mg/L); fasting plasma glucose levels (mg/dL); plasma ALT, AST, and γ-GTP levels (IU/L); abdominal circumference length (cm); red blood cell number (× 10^4^/μL) and blood Hb levels (g/dL); chest XP findings (A = normal, B = slight changes but no need for observation, C = need for observation, and H = need for treatment); ECG findings (A = normal, B = slight changes but no need for observation, C = need for observation, and H = need for treatment); work using visual display terminals (VDT) (A = need for observation, B = slight changes but no need for observation, and C = normal); interview regarding life habits (smoking at present: yes or no; more than 30 min exercise per day: yes or no; changes in body weight more than 2 kg over 1 year: yes or no; drinking alcohol at present: every day, not every day but sometimes, or none); and prescription details. We carefully performed data cleaning for all data. People were monitored for HF until 2014. HF was diagnosed by cardiologists and general practitioners using the Framingham Criteria of Congestive Heart Failure^11^, plasma BNP levels^12^ and echocardiogram^13^, which seems to be reliable to precisely and accurately diagnose the several types of HF.

We separated people with and without the occurrence of HF over 4 years (Table 1).

**Table 1:** Clinical characteristics of test and validation cohorts at baseline with or without the occurrence of heart failure in 4 years.

Variables	The cohort for LAMP analyses			The validation cohort (N = 275,658)
	Total (N = 32,547)	Without heart failure (N = 32,222)	With heart failure (N = 325)	
Women, n (%)	11,359 (34.9%)	11,272 (34.9%)	87 (24.7%)	75,009 (27.2%)
Age, median age	45 (36–52)	45 (36–52)	56 (46–64)*	54 (46–61)
BMI, median BMI	22.2 (20.2–24.4)	22.1 (20.2–24.3)	23.2 (21.3–25.7)*	22.3 (20.4–24.6)
Abd circumference, median cm	80.0 (74.0–86.0)	80.0 (74.0–86.0)	84.5 (78.5–90.5)*	81.0 (75.0–87.0)
sBP, median mmHg	117.0 (106.0–128.0)	116.0 (106.0–128.0)	126.0 (111.0–137.3)*	120.0 (110.0–131.0)
dBP, median mmHg	71.0 (64.0–80.0)	71.0 (64.0–80.0)	77.0 (68.0–86.0)*	74.0 (66.0–82.0)
Hb levels, median g/dl	14.4 (13.4–15.3)	14.4 (13.4–15.3)	14.7 (13.7–15.6)	14.7 (13.7–15.5)
plasma HbA1c levels, median %	5.2 (5.0–5.5)	5.2 (5.0–5.5)	5.5 (5.1–5.8)*	5.5 (5.2–5.7)
plasma HDL-cholesterol levels, median mg/dl	62.0 (53.0–74.0)	62.0 (53.0–74.0)	59.0 (50.0–71.0)*	60.0 (51.0–72.0)
plasma LDL-cholesterol levels, median mg/dl	120.0 (99.0–141.0)	120.0 (99.0–141.0)	125.0 (105.0–148.0)*	117.0 (96.0–139.0)
plasma TG levels, median mg/dl	86.0 (60.0–129.0)	86.0 (60.0–129.0)	105.0 (76.0–149.0)*	89.0 (62.0–134.0)
plasma AST levels, median IU/l	21.0 (18.0–25.0)	21.0 (18.0–25.0)	22.0 (19.0–26.0)	20.0 (17.0–24.0)
plasmaALT levels, median IU/l	19.0 (14.0–28.0)	19.0 (14.0–28.0)	21.0 (16.0–30.0)	19.0 (14.0–27.0)
plasma γGTP levels, median IU/dl	25.0 (17.0–42.0)	25.0 (17.0–42.0)	29.0 (21.0–50.0)*	25.0 (17.0–41.0)
plasma UA levels, median mg/dl	5.7 (4.7–6.6)	5.7 (4.7–6.6)	6.0 (5.1–7.0)*	5.4 (4.4–6.3)

BMI: body mass index, Abd circum: abdominal circumference, sBP: systolic blood pressure, dBP: diastolic blood pressure, Hb: hemoglobin, HbA1c: hemoglobin A1c, HDL: high density lipoprotein, LDL: low density lipoprotein, TG: triglyceride, AST: aspartate aminotransferase, ALT: alanine aminotransferase, γGTP: γ-glutamyl transpeptidase, UA: Uric Acid.

Values are median (interquartile range), only values of "women" are number (percent).

*P < 0.05 compared with the group without heart failure.

We employed the novel LAMP method for our data-mining analysis to identify rules consistent with single factors or combinations of factors that significantly affected the occurrence of cardiovascular events^8^. A person was represented by both individual clinical factors and the class labels of groups with or without the occurrence of HF, and this set of populations was used to form a data table in which each row represented a person. This data table D consisted of N rows, each of which consisted of M factors and a positive or negative class label for each object. LAMP uses Fisher’s exact tests to draw conclusions from a complete set of statistically significant hypotheses regarding a class label. Here, the hypothesis was based on a combination of class labels and conditions defined as a subset of the M factors in D. As the condition of the uncovered significant hypothesis may include any number of factors from 1 to M, the term “limitless-arity” has been used to describe this method. Accordingly, LAMP applies a highly efficient search algorithm to quickly and completely derive significant hypotheses from 2^M^ candidates.

If k is the number of all hypotheses for which the conditions exceed or remain equal to σ objects in D (σ < N), the relationship between k and σ (k = k_D_ (σ)) depends on D but is always antimonotonic because fewer hypothesis conditions remain true at a higher frequency of D. Although the formula of k_D_ (σ) is not analytically determined, LAMP includes a mining algorithm to efficiently derive all k hypothesis conditions under a given σ. Bonferroni correction sets a boundary for the familywise error rate of the false negatives in multiple tests at less than 1 significance level α by correcting the level to α/k_D_ (σ). Bonferroni correction can be used as a standard multiple-testing procedure for the k hypotheses. Note that this level is monotonic to σ as k_D_ (σ) is antimonotonic. If we use a very small set value for σ for a complete search of the significant hypotheses, α/k_D_ (σ) is extremely small because k_D_ (σ) approaches 2^M^. In this scenario, almost no hypotheses will be accepted as significant. Conversely, if the set values of σ and, consequently, α/k_D_ (σ) are too large, k_D_ (σ) will be very small and some significant hypothesis conditions will be missed. To overcome this limitation in LAMP, any hypothesis with a frequency less than σ will not have a *P* value less than the following level.

$$ f(\sigma ) = {{\left( {\begin{array}{*{20}c} {n_{p} } \\ \sigma \\ \end{array} } \right)} \mathord{\left/ {\vphantom {{\left( {\begin{array}{*{20}c} {n_{p} } \\ \sigma \\ \end{array} } \right)} {\left( {\begin{array}{*{20}c} N \\ \sigma \\ \end{array} } \right)}}} \right. \kern-0pt} {\left( {\begin{array}{*{20}c} N \\ \sigma \\ \end{array} } \right)}}. $$

Here, n_p_ is the number of objects with positive class labels in D (n_p_ < N). Accordingly, any hypothesis with a frequency less than σ will not be accepted if f(σ) > α/k_D_ (σ). Because f(σ) is antimonotonic for σ and α/k_D_ (σ) is monotonic, LAMP selects σ* to balance f(σ*) and α/k_D_ (σ*). The selected value of σ* yields the smallest number of candidate hypotheses without applying the tests or missing any significant hypotheses.

For practical reasons, we were interested in a hypothesis that held true for at least 10 people. As all hypotheses involving more than four factors failed to meet this criterion, we limited our LAMP-based search to a maximum of four factors. This limitation further reduced the number k_D_ (σ*) of the candidate hypotheses and increased the level α/k_D_ (σ*) in LAMP. After all significant hypotheses regarding single clinical factors or combinations of factors were obtained, we excluded each hypothesis for which the condition was a superset of conditions from other simpler hypotheses as the significance of the former would be trivial in comparison with the significance of the latter.

### Protocol 2

For a larger cohort of 275,658 general people, the clinical characteristics in 2010 were investigated (Table 1) and the occurrence of HF until 2014 was determined. The number of combinations of factors matching the predictive combinations of factors for the onset of HF obtained in Protocol 1 in each of the 275,658 people was determined. To prove the idea that the onset of HF is predictable using clinical variables, we tested the hypothesis that the number of combinations of factors matching the predictive combinations of factors for 2010 is linked to the actual occurrence of HF over 4 years. In detail, we checked how many combinations of factors for the prediction of the onset of HF discovered in Protocol I in each of individuals in Protocol II, and we classified 275,658 people of Protocol II into six groups who had 0, 1–50, 51–100, 101–150, 151–200 or 201–250 predictive combinations of factors discovered in Protocol 1 for the onset of HF.

It is a potentially informative censoring due to death from cardiovascular and non-cardiovascular deaths in this study, however this study did not analyze these clinical outcomes because no information of cardiovascular or non-cardiovascular death in the present data set is available.

### Statistical analyses

Descriptive statistics of continuous variables are presented as means with standard deviations. We tested the significant levels of all of the combinations of factors with no more than four clinical mutable variables among 288 variables observed in the present study, and LAMP automatically created multiple comparisons of 470,700 t-tests. We corrected the significance *P* value using Bonferroni correction in Protocol 1: We multiplied the *P* values by 470,700 and we used the multiplied *P* values for statistical analysis. In Protocol 2, since LAMP tests a significant combination based on the binarized data of 0 or 1, we used the Cochran's Q test, one of the nonparametric methods to perform multiple comparison to test whether the probability of the onset of HF increased as the number of combinations of factors matching the predictive combinations of factors increased. We also performed the Kaplan–Myer Analysis to test the time dependency of the results in Protocol 2. All *P* values were two-sided, and a *P* value of < 0.05 was considered statistically significant.

## Results

Table 1 lists patient clinical characteristics according to the development of HF. The clinical variables from 2010 were significantly different between the groups with and without HF. A LAMP analysis that maintained the familywise error rate below the required significance level by calibrating the Bonferroni factor to examine the significant combinations of factors of the 288 clinical variables (Supplementary Table 1) was performed, and thus, it characterized the HF outcomes. As shown in Table 1, the HF events occurred in 325 people among 32,547 people. In our LAMP analysis, we identified 549 combinations of factors that predicted the occurrence of HF (Supplementary Table 2). Interestingly, combinations of factors of single determinants for the onset of HF included age, systolic and diastolic blood pressure, and the use of aspirin or rosuvastatin. The other 544 combinations of factors consisted of two or three clinical factors.

**Figure 1:**
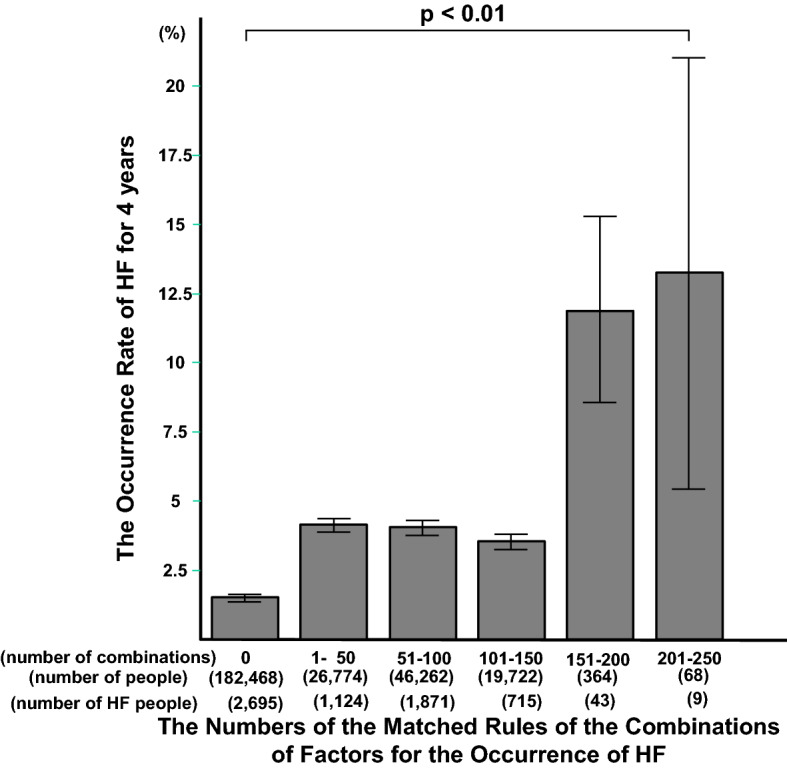
The relationship between the number of combinations of factors obtained in one cohort and the probability of HF occurrence in another cohort. As the number of the combinations of factors applied to each person increases, the probability of the incidence of HF increases.

To determine if the combinations of factors identified in the cohort of 32,547 people truly predicted HF, we analyzed another cohort of 275,658 people and determined if people with more predictive combinations of factors were more prone to developing HF. As the predictive combinations of factors that applied to a person increased, the incidence of HF increased (*P* < 0.01) over 4 years (Fig. 1). The numbers of the people for six groups who had 0, 1–50, 51–100, 101–150, 151–200 or 201–250 predictive combinations of factors discovered in Protocol 1 for the onset of HF were 182,468, 26,774, 46,262, 19,722, 364 and 68, respectively; the numbers of people with onset of HF for six groups were 2,695, 1,124, 1,871, 715, 43 and 9, respectively. Kaplan-Meyer Analysis showed that the incidence of HF time-dependently increased as the number of predictive combinations of factors increased (Supplementary Fig. 1). The probabilities for the HF occurrence in the groups with 0 and 201–250 were 1.48 and 13.2%, respectively: no combinations of factors to predict the HF occurrence in the low risk group really provided a very low probability of the incidence of the HF onset, and the risk for the onset of HF increases as the number of the combination for the onset of HF increased. On the other hand, we should notice the relatively low sensitivity for the detection of people of the highest risk groups caused HF in 4 years.

## Discussion

The key messages of the present investigation are twofold. First, this study provided evidence supporting the use of an AI model for identifying combinations of factors of clinical factors for predicting HF. For this purpose, we employed a novel data-mining method, LAMP, and big clinical data from the general population. Second, people who had more combinations of factors of clinical factors predicting HF were more likely to develop HF. The latter evidence strengthens the likelihood of the former evidence. This AI analysis can potentially be used for risk stratification for the occurrence of HF in general populations without HF.

### The novel strategy of mathematical evaluation for data-centric medicine

The present study proposed the expediency of data-mining of big data based on the LAMP^8^ to identify unexpected rules consisting of combinations of factors predicting the occurrence of HF. Data-mining methods are used to examine all possible combinations of factors of ordinary clinical variables that might or might not affect HF^14,15^. This approach allowed us to test both single variables and combinations of variables obtained at ordinary healthcare check-ups and from medical records of diseases that may not be directly linked to HF.

The LAMP method resembles a multivariate analysis. However, multivariate analyses only evaluate the effects of each variable on clinical outcomes and cannot determine the effects of combinations of factors. Although ordinary data-mining methods encounter a combinatorial explosion, LAMP maintains a statistical power under multiple comparisons and provides the significant *P* values for each factor against the outcomes with minimal false negatives by calibrating the Bonferroni factor. Importantly, single determinants for the onset of HF were age, systolic and diastolic blood pressure, and the use of aspirin or rosuvastatin (Table 1), and the other 544 combinations of factors consisted of two or three clinical factors, which have not been identified as potential risks for the onset of HF.

The LAMP method which provides the single or combination risk factors for HF is also different from the machine learning. The prediction of the onset of HF can be performed using machine learning such as random forest method and support vector machine^3,4^, however, the variables that determine the onset of HF was not clarified; if people are pointed out to be at high risk for HF, such analyses cannot point out the risks to be corrected. Furthermore, the time window for the prediction is relatively short.

The novel findings using LAMP with significant *P* value were also tested by the classical statistical methods such as Kaplan-Mayer Method. In the 1476 patients with old myocardial infarction (OMI) and glucose intolerance, we analyzed 415,328 combinations of factors of < 4 clinical parameters and identified 242 combinations of factors that predicted the occurrence of hospitalization and LAMP revealed that the use of proton pump inhibitors high plasma BNP levels, diuretics use, advanced age, and lack of anti-dyslipidemia drugs were linked to cardiovascular events, all of which were verified by independently drawn Kaplan–Meier curves, indicating that the determined factors accurately affected cardiovascular events^10^.

These were tested in another OMI cohort^16^ and HF cohort^9^. Therefore, the validity and reproducibility of LAMP were well verified in the cardiovascular field^17^.

### The importance of predicting the occurrence of HF

In a population-based study by Conrad et al.^18^, the incidence of HF declined by 7% between 2002 and 2014 from 3.6 to 3.3/1000 person-years, however the estimated absolute number of individuals with newly diagnosed heart failure in the UK increased by 12% largely due to an increase in population size and age; the estimated absolute number of prevalent HF cases in the UK increased even more by 23%. In the several hundred thousand people in our study, the annual HF incidence rate was approximately 2.5/1000 person-years, similar to the report of Conrad et al.^18^. The disparity of the rate of HF occurrence may partly attributable to HF diagnosis. The cardiologists have diagnosed HF using echocardiogram, CT/MRI examination, and blood test including the measurements of plasma BNP levels on the top of the physical examination and medical interview, and Framingham Criteria of Congestive Heart Failure^11^. However, general physicians may diagnose HF only by the symptom and physical examination, which may decrease the incidence of HF. In our data of Japan, HF is mainly diagnosed using Framingham Criteria of Congestive Heart Failure^11^, plasma BNP levels^12^ and echocardiogram^13^, which seems to be reliable to precisely and accurately diagnose the several types of HF.

Although mortality attributable to HF has improved over the past few decades, HF still has high 5-year mortality that rivals that of many cancers, and the prevalence of HF is increasing because of the aging population and increased risk factors, such as coronary artery disease, hypertension, smoking, obesity, and T2D^19^. Ho et al.^20^ showed that both hypertension and coronary heart disease are the most common conditions predating the onset of HF. T2D and electrocardiographic left ventricular hypertrophy are also associated with an increased risk of HF. Metabolic syndrome, or its components such as hypertension, dyslipidemia, and diabetes, have been recognized as independent potential risk factors for HF^21^. The prevalence of these factors seems to have decreased in the U.S. and Europe but has seemingly increased in Asia, especially in Japan. To respond to this unfavorable trend, the Japanese government launched the National Health Promotion Movement in the 21st Century (Health Japan 21) between 2001 and 2012. For > 10 years, this movement decreased the prevalence of high blood pressure only, while other factors remained unchanged or even worsened. As results, attempts to decrease the incidence of HF have been in vain. These lines of evidence hint that we need to treat the risks of HF, and we also need to identify the high-risk people among the general populations and provide the caution of the possibility of the onset of HF to provoke the behavior modulation. The present study may contribute to identify the risk people of HF; when such people are identified, we should exclude the risks for HF in each person to protect such a person from HF.

### Interpretation of the significant combinations of factors in predicting HF occurrence

HF is caused by multifactorial pathways, such as hypertension, T2D, and coronary artery diseases, and we assume that combinations of factors independently prime and/or cause the pathophysiology of HF. The present study demonstrated that 549 combinations of factors tightly and independently contribute to the high probability of HF occurrence. Each combination of factors significantly and independently causes HF due to the multiplexity of HF pathology, and each combination of factors seems to be an independent risk factor for the occurrence of HF. Individuals with more combinations are more likely to develop HF. Each combination not only contains the classic risk factors for HF but also contains factors, such as no T2D, no alcohol intake, no liver dysfunction and no smoking, that are considered inhibitory for HF. However, even if such factors are contained in the combinations, such combinations contain other deleterious factors that contribute to the high probability of developing HF. Alternatively, the combinations may phenomenologically or practically represent the high occurrence of HF, and the combinations may not guarantee a cause-and-effect relationship with HF. Importantly, people with more combinations are more prone to developing HF in a LAMP combination number-dependent manner, and this observation, conversely, suggests the multiplexity of causes of HF.

It is intriguing to know that the number of the predictive combinations determine the occurrence of HF with non-linear fashions (Fig. 1 and Supplementary Fig. 1). The accumulation of more than 1 or 150 risk factors increases the probability of the occurrence of HF with a stepwise fashion, suggesting that we need to further investigate how the combinations of the predictive LAMP determine the occurrence of HF.

## Conclusion

In conclusion, we were able to identify combinations of clinical variables that predict the new-onset HF in the general population, and more combinations proportionally increased the probability of HF onset in the general population. This quantitative AI method can be used to stratify the probability of developing HF and identify the high-risk cohort for new-onset HF in the general population.

## Supplementary Information

Supplementary Figure 1.

Supplementary Table 1.

Supplementary Table 2.

## Data Availability

The data analyzed in this study are available from the corresponding author upon reasonable request.

## References

[1] Tsutsui H, Tsuchihashi-Makaya M, Kinugawa S, Goto D, Takeshita A (2007). Characteristics and outcomes of patients with heart failure in general practices and hospitals. Circ. J..

[2] Sahle BW, Owen AJ, Chin KL, Reid CM (2017). Risk prediction models for incident heart failure: a systematic review of methodology and model performance. J. Card. Fail..

[3] Wang Y, Ng K, Byrd RJ (2015). Early detection of heart failure with varying prediction windows by structured and unstructured data in electronic health records. Annu. Int. Conf. IEEE Eng. Med. Biol. Soc..

[4] Wu J, Roy J, Stewart WF (2010). Prediction modeling using EHR data: challenges, strategies, and a comparison of machine learning approaches. Med. Care.

[5] Choi E, Schuetz A, Stewart WF, Sun J (2017). Using recurrent neural network models for early detection of heart failure onset. J. Am. Med. Inform. Assoc..

[6] Segar MW, Jaeger BC, Patel KV (2021). Development and validation of machine learning-based race-specific models to predict 10-year risk of heart failure: A multicohort analysis. Circulation.

[7] Khan SS, Ning H, Shah SJ (2019). 10-Year risk equations for incident heart failure in the general population. J. Am. Coll. Cardiol..

[8] Terada A, Okada-Hatakeyama M, Tsuda K, Sese J (2013). Statistical significance of combinatorial regulations. Proc. Natl. Acad. Sci. U.S.A..

[9] Fukuda H, Shindo K, Sakamoto M (2018). Elucidation of the strongest predictors of cardiovascular events in patients with heart failure. EBioMedicine.

[10] Shindo K, Fukuda H, Hitsumoto T (2020). Artificial intelligence uncovered clinical factors for cardiovascular events in myocardial infarction patients with glucose intolerance. Cardiovasc. Drugs Ther..

[11] McKee PA, Castelli WP, McNamara PM, Kannel WB (1971). The natural history of congestive heart failure: the Framingham study. N. Engl. J. Med..

[12] McMurray JJ, Adamopoulos S, Anker SD (2012). ESC Guidelines for the diagnosis and treatment of acute and chronic heart failure 2012: The Task Force for the Diagnosis and Treatment of Acute and Chronic Heart Failure 2012 of the European Society of Cardiology. Developed in collaboration with the Heart Failure Association (HFA) of the ESC. Eur. Heart J..

[13] Ohte N, Ishizu T, Izumi C (2022). JCS 2021 guideline on the clinical application of echocardiography. Circ. J..

[14] Podgorelec V, Kokol P, Stiglic B, Rozman I (2002). Decision trees: an overview and their use in medicine. J. Med. Syst..

[15] Kim J, Washio T, Yamagishi M (2004). A novel data mining approach to the identification of effective drugs or combinations for targeted endpoints–application to chronic heart failure as a new form of evidence-based medicine. Cardiovasc. Drugs Ther..

[16] Shindo K, Fukuda H, Hitsumoto T (2020). Plasma BNP levels and diuretics use as predictors of cardiovascular events in patients with myocardial infarction and impaired glucose tolerance. Cardiovasc. Drugs Ther.

[17] Kitakaze M, Asakura M, Nakano A, Takashima S, Washio T (2015). Data mining as a powerful tool for creating novel drugs in cardiovascular medicine: the importance of a "back-and-forth loop" between clinical data and basic research. Cardiovasc. Drugs Ther..

[18] Conrad N, Judge A, Tran J (2018). Temporal trends and patterns in heart failure incidence: A population-based study of 4 million individuals. Lancet.

[19] Bui AL, Horwich TB, Fonarow GC (2011). Epidemiology and risk profile of heart failure. Nat. Rev. Cardiol..

[20] Ho Kalon KL, Pinsky Joan L, Kannel William B, Levy D (1993). The epidemiology of heart failure: The Framingham Study. J. Am. Coll. Cardiol..

[21] Zimmet P, Magliano D, Matsuzawa Y, Alberti G, Shaw J (2005). The metabolic syndrome: A global public health problem and a new definition. J. Atheroscler. Thromb..

