# Supplementary material for: Predicting heart failure onset in the general population using a novel data-mining artificial intelligence method
Source: Sci Rep. 2023 Mar 16;13:4352. doi: 10.1038/s41598-023-31600-0 (PMC10020464; doi:10.1038/s41598-023-31600-0)
Supplement: Supplementary file 1 — Supplementary Figure 1. [file 41598_2023_31600_MOESM1_ESM.pdf]

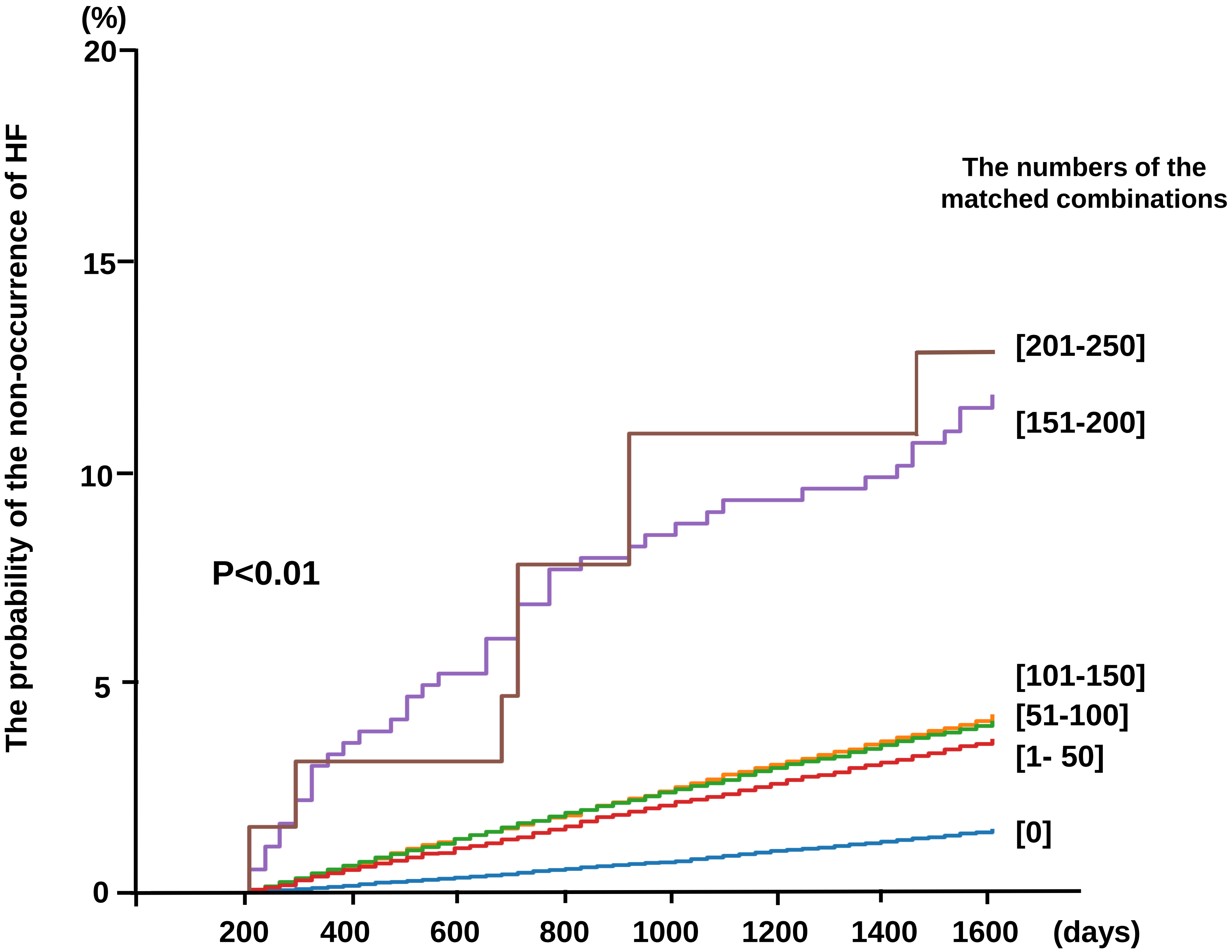

Supplementary Figure 1. Kaplan-Meyer Analysis for HF occurrence in the 6 groups classified by the number of the combinations of factors applied to each person.
