# Supplementary material for: Predicting heart failure onset in the general population using a novel data-mining artificial intelligence method
Source: Sci Rep. 2023 Mar 16;13:4352. doi: 10.1038/s41598-023-31600-0 (PMC10020464; doi:10.1038/s41598-023-31600-0)
Supplement: Supplementary file 2 — Supplementary Table 1. [file 41598_2023_31600_MOESM2_ESM.pdf]

Supplementary Table 1. The numerical values and the prevalence of valuables of the clinical factorswith and without occurrence of heart failure

	peope without occurerence of heart failure (N=32,222)			people with occurerence of heart failure (N=325)		
**Variables**	**25th percentile**	**mean**	**75th percentile**	**25th percentile**	**mean**	**75th percentile**
**Numerical Variables**						
age at the end of the year	46	56	63	36	44	52
age at March	46	56	64	36	45	52
time after dinner	2.25	3.25	4.625	2	3	5.5
height	161.0	166.7	172.7	160.6	167.6	173.2
body weight	57.2	65.7	74.8	53.7	62.6	70.8
BMI	21.3	23.2	25.7	20.2	22.1	24.3
Abd circumference, cm	78.5	84.5	90.5	74	80	86
systolic BP for the 1st time (mmHg)	111	126	137	106	116	128
diastolic BP for the 1st time (mmHg)	68	77	86	64	71	80
systolic BP for the 2nd time (mmHg)	124	136	146	121	130	138
diastolic BP for the 2nd time (mmHg)	80	88	96	74	82	88
casual systolic BP (mmHg)	112	123	138	106	116	125
casual diastolic BP (mmHg)	68	78	87	64	71	79
plasma ALT levels (IU/L)	19	22	26	18	21	25
plasma AST levels (IU/L)	16	21	30	14	19	28
plasma γ-GTP levels (IU/L)	21	29	50	17	25	42
plasma triglyceride levels (mg/L)	76	105	149	60	86	129
plasma HDL cholesterol levels (mg/L)	50	59	71	53	62	74
plasma LDL cholesterol levels (mg/L)	105	125	148	99	120	141
plasma uric acid levels (mg/L)	5.1	6.0	7.0	4.7	5.7	6.6

fasting plasma glucose levels (mg/dL);	89	94	102	84	90	96
the plasma HbA1c levels（JDS）	4.8	5.1	5.4	4.7	4.9	5.1
plasma HbA1c levels（NGSP）	5.1	5.5	5.8	5	5.2	5.5
red blood cell number (×104/mL)	440	481	508	442	472	501
and blood Hb levels (g/dL)	13.7	14.7	15.6	13.4	14.4	15.3
naked eye acuity distance (right)	0.6	0.9	1.2	0.7	1	1.5
naked eye acuity distance (left)	0.6	0.85	1	0.7	1	1.5
corrected eye acuity distance (right)	0.9	1.2	1.2	0.9	1	1.2
corrected eye acuity (left)	0.9	1	1.2	0.9	1	1.2
naked eye acuity at 50cm distance (right)	0.78	1	1.2	0.9	1.2	1.5
naked eye acuity at 50cm distance (left)	0.9	1	1.2	0.9	1.2	1.5
corrected eye acuity at 50cm distance (right)	0.8	1	1.2	0.9	1.2	1.2
corrected eye acuity at 50cm distance (left)	0.8	1	1.2	0.9	1.2	1.2
The age of onset of hypertention	42.0	45.5	51.0	40.0	45.0	50.0
the age of oset of dyslipidemia	40.0	46.5	50.5	36.0	43.0	48.0
the age of onset of diabetes mellitus	36.5	44.5	48.5	40.0	45.0	50.0
the age of onset of hyperuricemia or goat	39.5	44.5	46.3	35.0	41.0	47.0
the age of onset of pulmonary tubercurosis		none		18.0	24.0	30.0
the age of onset of bronchial asthma	29.0	42.5	49.0	7.0	16.0	34.0
the age of onset of respiratory disease	58.0	58.0	58.0	22.8	35.0	42.0
the age of onset of angina pectoris or myocardial infarction	47.0	48.0	58.0	38.0	45.0	50.0
the age of onset of arrhythmia	34.3	42.5	46.3	22.5	36.0	45.0
the age of onset of cerebrovascular diasease	44.0	44.0	44.0	36.0	43.0	48.8
the age of onset of anemia	31.0	45.0	46.0	19.0	29.0	38.0

the age of onset of hematologic disease	40.0	40.0	40.0	26.0	35.0	45.0
the age of onset of liver dysfunction	37.3	43.0	48.0	30.0	39.0	45.0
the age of onset of hepatitis B	36.3	37.5	38.8	20.0	27.0	33.0
the age of onset of hepatitis C	23.0	23.0	23.0	26.8	35.5	42.0
the age of onset of renal disease	30.5	46.0	47.0	10.0	17.5	31.3
the age of onset of urinary stone	36.0	40.0	41.0	30.0	38.5	45.0
the age of onset of gallballader stone or polyp	39.5	46.0	50.0	34.0	40.0	45.0
the age of onset of pancreatitis		none		25.0	31.0	42.3
the age of onset of rheumatoid arthritis		none		24.0	36.5	44.8
the age of onset of gastric ulcer	24.3	30.0	44.5	26.0	34.0	40.8
the age of onset of gastric polyp	41.0	47.0	50.0	35.0	41.0	46.0
the age of onset of colon polyp	41.0	44.0	50.0	40.0	45.0	50.0
the age of onset of hemorrhoids	30.0	35.0	40.0	29.0	35.0	42.0
the age of onset of other gastrointestical diseases	46.3	47.5	48.8	30.0	39.0	46.0
the age of onset of benign prostatic hyperplasia	46.5	50.0	54.5	48.0	52.0	57.0
the age of onset of thyroid diseases	35.0	35.0	35.0	26.0	34.0	42.0
the age of onset of myoma uteri	40.5	45.0	46.0	34.0	38.0	43.0
the age of onset of cataracta		none		36.0	45.0	53.0
the age of onset of glaucoma	46.5	49.0	51.5	37.0	43.0	49.0

Categolized Variables	applicable cases	non-applicable cases	applicable cases	non-applicable cases
women	11272		87	
men	20950		238	
urinary sugar levels (borderline)	56	32166	0	325
urinary sugar levels (1+)	156	32066	1	324
urinary sugar levels (2+)	90	32132	0	325
urinary sugar levels (3+)	110	32112	1	324
urinary sugar levels (4+)	7	32215	0	325
urinary protein levels (borderline)	1008	31214	20	305
urinary protein levels (1+)	741	31481	11	314
urinary protein levels (2+)	196	32026	5	320
urinary protein levels (3+)	66	32156	5	320
urinary protein levels (4+)	9	32213	0	325
chest XP findings (A = normal)	22406	9816	163	162
chest XP findings (B = slight changes but no need for observation)	634	31588	9	316
chest XP findings (C = need for observation)	122	32100	3	322
chest XP findings (H = need for treatment)	1	32221	0	325
chest XP findings (H1 = need for urgent treatment)	2	32220	0	325
chest XP findings (H2 = need for precise examination)	3	32219	1	324
ECG findings (A = normal)	21889	10333	140	185
ECG findings (B = slight changes but no need for observation)	1181	31041	15	310

ECG findings (C = need for observation)	237	31985	11	314
ECG findings (H = need for treatment)	90	32132	6	319
ECG findings (H1 = need for urgent treatment)	14	32208	1	324
ECG findings (H2 = need for precise examination)	29	32193	3	322
VDT (A = need for observation)	17254	14968	133	192
VDT (B = slight changes but no need for observationl)	5571	26651	39	286
VDT(C = normal)	605	31617	4	321
smoking at present: no	26387	5835	269	56
smoking at present: yes	5701	26521	54	271
more than 30 min exercise per day: no	23028	9194	199	126
more than 30 min exercise per day: yes	6677	25545	75	250
changes in body weight more than 2 kg over 1 year: no	21030	11192	181	144
changes in body weight more than 2 kg over 1 year: yes	8666	23556	92	233
drinking alcohol at present: not every day but sometimes	13659	18563	112	213
drinking alcohol at present: none	8266	23956	78	247
drinking alcohol at present: every day	7780	24442	84	241
the tretament with nifedipine	95	32127	5	320
the treatment with amlodipine	461	31761	16	309
the treatment with valsartan amlodipine besilate	51	32171	3	322
the treatment with omlmesartan medoxomil	274	31948	12	313
the treatment with benidipine	83	32139	7	318

the treatment with valsartan	365	31857	12	313
the treatment with candesartan cilexetil	318	31904	7	318
the treatment with telmisartan	241	31981	9	316
the treatment with ethyl Icosapentate	81	32141	4	321
the treatment with rosuvastatin	505	31717	21	304
the treatment with ezetimibe	134	32088	5	320
the treatment with pravastatin sodium	126	32096	3	322
the treatment with pitavastatin calcium hydrate	251	31971	11	314
the treatment with atorvastatin calcium hydrate	453	31769	13	312
the treatment with glimepiride	174	32048	7	318
the treatment with metformin hydrochloride	177	32045	8	317
the treatment with sitagliptin phosphate hydrate	137	32085	1	324
the treatment with vildagliptin	32	32190	0	325
the treatment with levofloxacin hydrate	2654	29568	31	294
the treatment with clarithromycin	1020	31202	8	317
the treatment with amoxicillin hydrate	318	31904	6	319
the treatment with azithromycin hydrate	1587	30635	15	310
the treatment with cefdinir	669	31553	5	320
the treatment with cefditoren pivoxil	1415	30807	16	309
the treatment with oseltamivir phosphate	375	31847	5	320

the treatment with cefcapene pivoxil hydrochloride hydrate	2628	29594	32	293
the treatment with valaciclovir hydrochloride	381	31841	7	318
the treatment with garenoxacin mesilate hydrate	1067	31155	15	310
the treatment with fexofenadine hydrochloride	2486	29736	28	297
the treatment with epinastine hydrochloride	561	31661	9	316
the treatment with olopatadine hydrochloride	269	31953	2	323
the treatment with loratadine	372	31850	4	321
the treatment with cetirizine hydrochloride	456	31766	2	323
the treatment with d -chlorpheniramine maleate	673	31549	8	317
the treatment with betamethasone /d -chlorpheniramine maleate	1098	31124	12	313
the treatment with bepotastine besilate	265	31957	1	324
the treatment with pranlukast hydrate	439	31783	8	317
the treatment with levocetirizine hydrochloride	725	31497	4	321
the treatment with mequitazine	374	31848	3	322
the treatment with dimethicone	6	32216	0	325
the treatment with rabeprazole sodium	415	31807	12	313
the treatment with famotidine	562	31660	15	310
the treatment with mosapride citrate hydrate	607	31615	13	312

the treatment with antibiotics-resistant lactic acid bacteriae	1019	31203	14	311
the treatment with scopolamine butylbromide	505	31717	5	320
the treatment with metoclopramide hydrochloride	280	31942	4	321
the treatment with sennoside	126	32096	6	319
the treatment with L-glutamine	218	32004	2	323
the treatment with rebamipide	1605	30617	28	297
the treatment with teprenone	1092	31130	14	311
the treatment with lansoprazole	343	31879	12	313
the treatment with magnesium oxide	174	32048	4	321
the treatment with esomeprazole magnesium hydrate	83	32139	1	324
the treatment with sodium picosulfate hydrate	102	32120	3	322
the treatment with sulpiride	252	31970	3	322
the treatment with sodium hydrogen carbonate	35	32187	0	325
the treatment with sennoside A/B	112	32110	0	325
the treatment with domperidone	713	31509	5	320
the treatment with hydrocortisone	205	32017	3	322
the treatment with magnesium citrate	23	32199	2	323
the treatment with berberine chloride hydrate	323	31899	6	319
the treatment with cefmenoxime hydrochloride	12	32210	0	325
the treatment with ofloxacin otic solution	165	32057	4	321

the treatment with fosfomycin sodium	16	32206	0	325
the treatment with mometasone furoate hydrate nasal slution	913	31309	13	312
the treatment with fluticasone furoate nasal solution	686	31536	10	315
the treatment with adenosine triphosphate disodium hydrate	317	31905	3	322
the treatment with sodium cromoglicate nasal solution	59	32163	2	323
the treatment with ketotifen fumarate nasal solution	25	32197	1	324
the treatment with tramazoline hydrochloride nasal solution	194	32028	4	321
the treatment with ofloxacin ophthalmic ointment	191	32031	3	322
the treatment with gatifloxacin hydrate ophthalmic solution	239	31983	4	321
the treatment with pirenoxine ophthalmic solution	187	32035	7	318
the treatment with levofloxacin hydrate ophthalmic solution	1005	31217	9	316
the treatment with fluorometholone ophthalmic solution	1102	31120	11	314
the treatment with dexamethasone sodium phosphate ophthalmic solution	32	32190	0	325
the treatment with betamethasone ophthalmic solution	218	32004	5	320
the treatment with jatanoprost ophthalmic solution	137	32085	3	322
the treatment with cyanocobalamin ophthalmic solution	280	31942	6	319

the treatment with diquafosol sodium ophthalmic solution	127	32095	2	323
the treatment with purified sodium hyaluronate ophthalmic solution	733	31489	11	314
the treatment with oxymetazoline hydrochloride ophthalmic solution	24	32198	1	324
the treatment with olopatadine hydrochloride ophthalmic solution	1525	30697	22	303
the treatment with tropicamide phenylephrine hydrochloride ophthalmic solution	17	32205	0	325
the treatment with levocabastine hydrochloride ophthalmic solution	713	31509	9	316
the treatment with acetaminophen	2621	29601	22	303
the treatment with diclofenac sodium	248	31974	7	318
the treatment with eperisone hydrochloride	496	31726	11	314
the treatment with loxoprofen sodium hydrate	5346	26876	62	263
the treatment with celecoxib	344	31878	11	314
the treatment with ketoprofen	1133	31089	26	299
the treatment with loxoprofen sodium hydrate	899	31323	15	310
the treatment with felbinac	265	31957	7	318
the treatment with oregabalin	124	32098	6	319
the treatment with flurbiprofen	308	31914	2	323
the treatment with lornoxicam	312	31910	5	320

the treatment with mefenamic acid	356	31866	2	323
the treatment with felbinac	189	32033	4	321
the treatment with lidocaine	11	32211	0	325
the treatment with naphazoline nitrate	156	32066	0	325
the treatment with adrenaline	4	32218	0	325
the treatment with febuxostat	15	32207	0	325
the treatment with allopurinol	255	31967	9	316
the treatment with benzbromarone	175	32047	5	320
the treatment with sodium citrate hydrate	90	32132	1	324
the treatment with clobetasone butyrate ointment	197	32025	0	325
the treatment with tacrolimus hydrate ointment	276	31946	2	323
the treatment withvaseline	522	31700	5	320
the treatment with difluprednate ointment	523	31699	4	321
the treatment with dexamethasone propionate ointment	183	32039	0	325
the treatment with betamethasone valerate /gentamicin sulfate lotion	194	32028	1	324
the treatment with dimethyl iIsopropylazulene ointment	242	31980	4	321
the treatment with betamethasone butyrate propionate lotion	168	32054	1	324
the treatment with heparinoid ointment	896	31326	11	314

the treatment with hydrocortisone butyrate ointment	453	31769	3	322
the treatment with nadifloxacin cream	225	31997	3	322
the treatment with gentamicin sulfate ointment	645	31577	9	316
the treatment with clindamycin phosphate gel	274	31948	1	324
the treatment with clobetasol propionate ointment	268	31954	9	316
the treatment with ketoconazole cream	251	31971	3	322
the treatment with fradiomycin sulfate /methylprednisolone ointment	333	31889	5	320
the treatment with heparinoid cream	130	32092	1	324
the treatment with prednisolone valerate acetate ointment	219	32003	4	321
the treatment with luliconazole cream	224	31998	2	323
the treatment with saline	13	32209	0	325
the use of distilled water for injection	1	32221	0	325
the use of sterile purified water	32	32190	0	325
the treatment with modified merry life powder	86	32136	0	325
the treatment pueraria decoction	362	31860	3	322
the treatment with minor blue dragon decoction	306	31916	2	323
the treatment with major middle-strengthening decoction	74	32148	2	323
the treatment with angelica and peony powder	103	32119	0	325
the treatment with dwarf lilyturf decoction	412	31810	4	321

the treatment with middle-reinforcing and qi-benefiting decoction	108	32114	1	324
the treatment with rikkunshito	97	32125	3	322
the treatment with peony and licorice decoction	134	32088	4	321
the treatment with salicylamide/ acetaminophen/anhydrous	2074	30148	23	302
the treatment with dequalinium chloride	1196	31026	14	311
the use of sodium gualenate hydrate	1118	31104	13	312
the treatment with ambroxol hydrochloride	1183	31039	15	310
the treatment with L-carbocisteine	3510	28712	41	284
the treatment with dextromethorphan hydrobromide hydrate	1921	30301	17	308
the treatment with eprazinone hydrochloride	500	31722	5	320
the treatment with dimemorfan phosphate	546	31676	6	319
the treatment with tipepidine hibenzate	994	31228	8	317
the treatment with povidone-Iodine gargle solution	682	31540	12	313
the treatment with laninamivir octanoate hydrate	257	31965	2	323
the treatment with ibuprofen	959	31263	12	313
the treatment with dihydrocodeine phosphate /dl -methylephedrine	1109	31113	12	313
the treatment with cloperastine fendizoate	252	31970	6	319
the treatment with benproperine phosphate	406	31816	1	324
the treatment with aspirin	255	31967	25	300

the treatment with clopidogrel sulfate	71	32151	5	320
the treatment with warfarin potassium	77	32145	13	312
the treatment with salmeterol xinafoate /fluticasone propionate	463	31759	7	318
the treatment with pranlukast hydrate	511	31711	4	321
the treatment with montelukast sodium	588	31634	5	320
the treatment with tulobuterol	656	31566	7	318
the treatment with budesonide /formoterol fumarate hydrate	301	31921	7	318
the treatment with betamethasone sodium phosphate	2	32220	0	325
the treatment with prednisolone	526	31696	8	317
the treatment with zolpidem tartrate	560	31662	11	314
the treatment with brotizolam	126	32096	6	319
the treatment with etizolam	557	31665	8	317
the treatment with triazolam	151	32071	2	323
the treatment with clotiazepam	174	32048	3	322
the treatment with paroxetine hydrochloride hydrate	254	31968	3	322
	235	31987	5	320
the treatment with escitalopram oxalate	21	32201	0	325
the treatment with duloxetine hydrochloride	164	32058	1	324
the treatment with mirtazapine	110	32112	1	324

the treatment withlithium carbonate	67	32155	0	325
the treatment with lorazepam	127	32095	2	323
the treatment with bromazepam	127	32095	1	324
the treatment with alprazolam	217	32005	5	320
the treatment with ethyl loflazepate	176	32046	1	324
the treatment with clonazepam	100	32122	2	323
the treatment with aripiprazole	62	32160	1	324
the treatment with ursodeoxycholic acid	120	32102	4	321
the treatment with tranexamic acid	176	32046	3	322
the treatment with thiamazole	52	32170	1	324
the treatment with ascorbic acid /calcium pantothenate	437	31785	2	323
the treatment with mecobalamin	146	32076	3	322
the treatment with flavin adenine dinucleotide sodium	210	32012	2	323
the treatment with eldecalcitol	7	32215	1	324
the treatment with neurotropin	171	32051	5	320
the treatment with betahistine mesilate	236	31986	1	324
the treatment with sodium ferrous citrate	203	32019	0	325
the treatment with tyloxapol	3	32219	0	325

Abbreviations: ALT : alanine aminotransferase, AST : aspartate aminotransferase, γGTP : γ-glutamyl transpeptidase, LDL: low density lipoprotein, HbA1c : hemoglobin A1c, VDT: visual display terminals
