# Supplementary material for: Predicting heart failure onset in the general population using a novel data-mining artificial intelligence method
Source: Sci Rep. 2023 Mar 16;13:4352. doi: 10.1038/s41598-023-31600-0 (PMC10020464; doi:10.1038/s41598-023-31600-0)
Supplement: Supplementary file 3 — Supplementary Table 2. [file 41598_2023_31600_MOESM3_ESM.pdf]

Supplementary Table 2. The combinations of clinical factors that predicted the occurrence of heart failure

The combinations of clinical parameters			Adjusted p-value
The plasma AST levels no greater than 40IU/L	The age of 60 or more than 60 years old	The urinary glucose: Borderline levels	1.10E-26
The plasma ALT levels no greater than 40IU/L	The age of 60 or more than 60 years old	The urinary glucose: Borderline levels	6.06E-26
The plasma AST levels no greater than 40IU/L	The age of 60 or more than 60 years old		9.74E-26
The age of 60 or more than 60 years old	The urinary glucose: Borderline levels		1.78E-25
The plasma ALT levels no greater than 40IU/L	The plasma AST levels no greater than 40IU/L	The age of 60 or more than 60 years old	2.73E-25
The plasma ALT levels no greater than 40IU/L	The age of 60 or more than 60 years old		5.03E-25
The age of 60 or more than 60 years old			1.95E-24
The plasma HbA1c levels no greater than 6.2%	The plasma AST levels no greater than 40IU/L	The age of 60 or more than 60 years old	6.60E-24
The plasma ALT levels no greater than 40IU/L	The age of 60 or more than 60 years old	The plasma γ-GTP levels no greater than 71IU/L	8.82E-24
The age of 60 or more than 60 years old	The urinary glucose: Borderline	The plasma γ-GTP levels no greater than 71IU/L	1.03E-23
The plasma ALT levels no greater than 40IU/L	The plasma HbA1c levels no greater than 6.2%	The age of 60 or more than 60 years old	1.59E-23
The plasma AST levels no greater than 40IU/L	The age of 60 or more than 60 years old	The plasma γ-GTP levels no greater than 71IU/L	1.99E-23
The plasma HbA1c levels no greater than 6.2%	The age of 60 or more than 60 years old	The urinary glucose: Borderline levels	3.36E-23
The plasma HbA1c levels no greater than 6.2%	The age of 60 or more than 60 years old		6.20E-23
The age of 60 or more than 60 years old	The plasma γ-GTP levels no greater than 71IU/L		7.47E-23
The plasma HDL cholesterol levels more than 40mg/L	The plasma AST levels no greater than 40IU/L	The age of 60 or more than 60 years old	3.11E-22
The plasma HDL cholesterol levels more than 40mg/L	The age of 60 or more than 60 years old	The urinary glucose: Borderline levels	4.63E-22
The plasma ALT levels no greater than 40IU/L	The plasma HDL cholesterol levels more than 40mg/L	The age of 60 or more than 60 years old	4.98E-22
The plasma HDL cholesterol levels more than 40mg/L	The age of 60 or more than 60 years old		3.65E-21

The plasma HbA1c levels no greater than 6.2%	The age of 60 or more than 60 years old	The plasma γ-GTP levels no greater than 71IU/L	9.79E-21
The plasma HDL cholesterol levels more than 40mg/L	The plasma HbA1c levels no greater than 6.2%	The age of 60 or more than 60 years old	1.57E-20
The habitual smoking: No	The plasma AST levels no greater than 40IU/L	The age of 60 or more than 60 years old	5.00E-20
The habitual smoking: No	The age of 60 or more than 60 years old	The urinary glucose: Borderline levels	9.23E-20
The plasma ALT levels no greater than 40IU/L	The age of 60 or more than 60 years old	The fasting plasma glucose levels less than 110mg/dL	1.19E-19
The plasma AST levels no greater than 40IU/L	The age of 60 or more than 60 years old	The fasting plasma glucose levels less than 110mg/dL	1.74E-19
The plasma HDL cholesterol levels more than 40mg/L	The age of 60 or more than 60 years old	The plasma γ-GTP levels no greater than 71IU/L	2.10E-19
The habitual smoking: No	The plasma ALT levels no greater than 40IU/L	The age of 60 or more than 60 years old	3.66E-19
The age of 60 or more than 60 years old	The urinary glucose: Borderline	The fasting plasma glucose levels less than 110mg/dL	4.80E-19
The habitual smoking: No	The age of 60 or more than 60 years old	The urinary protein:Borderline	5.59E-19
The plasma AST levels no greater than 40IU/L	The age of 60 or more than 60 years old		7.67E-19
The age of 60 or more than 60 years old	The fasting plasma glucose levels less than 110mg/dL		8.88E-19
The gender: Male	The plasma AST levels no greater than 40IU/L	The age of 60 or more than 60 years old	1.22E-18
The habitual smoking: No	The plasma HbA1c levels no greater than 6.2%	The age of 60 or more than 60 years old	1.25E-18
The age of 60 or more than 60 years old	The urinary glucose: Borderline levels	The urinary protein:Borderline	1.76E-18
The gender: Male	The age of 60 or more than 60 years old	The urinary glucose: Borderline levels	1.86E-18
The plasma ALT levels no greater than 40IU/L	The gender: Male	The age of 60 or more than 60 years old	2.13E-18
The urinary protein:Borderline	The age of 60 or more than 60 years old	The plasma γ-GTP levels no greater than 71IU/L	4.52E-18
The plasma ALT levels no greater than 40IU/L	The age of 60 or more than 60 years old	The urinary protein:Borderline	5.71E-18
The plasma HbA1c levels no greater than 6.2%	The age of 60 or more than 60 years old	The fasting plasma glucose levels less than 110mg/dL	6.43E-18
The age of 60 or more than 60 years old	The urinary protein:Borderline		7.38E-18

The plasma γ-GTP levels no greater than 71IU/L,	The age of 60 or more than 60 years old	The fasting plasma glucose levels less than 110mg/dL	8.96E-18
The gender: Male	The age of 60 or more than 60 years old		1.68E-17
The plasma HDL cholesterol levels more than 40mg/L	The age of 60 or more than 60 years old	The fasting plasma glucose levels less than 110mg/dL	1.94E-17
The habitual smoking: No	The age of 60 or more than 60 years old	The plasma γ-GTP levels no greater than 71IU/L	1.98E-17
The gender: Male	The plasma HbA1c levels no greater than 6.2%	The age of 60 or more than 60 years old	5.21E-17
The gender: Male	The age of 60 or more than 60 years old	The plasma γ-GTP levels no greater than 71IU/L	3.39E-16
The plasma HbA1c levels no greater than 6.2%	The age of 60 or more than 60 years old	The urinary protein:Borderline	3.69E-16
The habitual smoking: No	The plasma HDL cholesterol levels more than 40mg/L	The age of 60 or more than 60 years old	3.84E-16
The plasma HDL cholesterol levels more than 40mg/L	The age of 60 or more than 60 years old	The urinary protein:Borderline	1.09E-15
The habitual smoking: No	The age of 60 or more than 60 years old	The fasting plasma glucose levels less than 110mg/dL	3.90E-15
The plasma LDL cholesterol levels less than 140 mg/dL	The plasma AST levels no greater than 40IU/L	The age of 60 or more than 60 years old	5.73E-15
The plasma AST levels no greater than 40IU/L	The age of 60 or more than 60 years old	The plasma triglyceride levels less than 150 mg/dL	6.53E-15
The urinary glucose: Borderline	The age of 60 or more than 60 years old	The plasma triglyceride levels less than 150 mg/dL	8.30E-15
The plasma LDL cholesterol levels less than 140 mg/dL	The age of 60 or more than 60 years old	The urinary glucose: Borderline levels	9.63E-15
The body mass index between 18.5-24.9	The age of 60 or more than 60 years old	The urinary glucose: Borderline levels	1.46E-14
The plasma ALT levels no greater than 40IU/L	The plasma LDL cholesterol levels less than 140 mg/dL	The age of 60 or more than 60 years old	1.81E-14
The plasma ALT levels no greater than 40IU/L	The age of 60 or more than 60 years old	The plasma triglyceride levels less than 150 mg/dL	1.81E-14
The body mass index between 18.5-24.9	The plasma AST levels no greater than 40IU/L	The age of 60 or more than 60 years old	1.83E-14
The age of 60 or more than 60 years old	The plasma triglyceride levels less than 150 mg/dL		3.10E-14

The plasma HDL cholesterol levels more than 40mg/L	The gender: Male	The age of 60 or more than 60 years old	3.65E-14
The plasma LDL cholesterol levels less than 140 mg/dL	The age of 60 or more than 60 years old		5.03E-14
The body mass index between 18.5-24.9	The plasma ALT levels no greater than 40IU/L	The age of 60 or more than 60 years old	6.66E-14
The body mass index between 18.5-24.9	The age of 60 or more than 60 years old		7.32E-14
The age of 60 or more than 60 years old	The plasma triglyceride levels less than 150 mg/dL,	The plasma γ-GTP levels no greater than 71IU/L	9.49E-14
The urinary protein:Borderline	The age of 60 or more than 60 years old	The fasting plasma glucose levels less than 110mg/dL	1.14E-13
The body mass index between 18.5-24.9	The age of 60 or more than 60 years old	The plasma γ-GTP levels no greater than 71IU/L	1.75E-13
The plasma HbA1c levels no greater than 6.2%	The systolic blood pressure between 80-139mmHg	The age of 60 or more than 60 years old	2.25E-13
The plasma AST levels no greater than 40IU/L	The systolic blood pressure between 80-139mmHg	The age of 60 or more than 60 years old	2.69E-13
The plasma LDL cholesterol levels less than 140 mg/dL	The age of 60 or more than 60 years old	The plasma γ-GTP levels no greater than 71IU/L	2.71E-13
The habitual smoking: No	The gender: Male	The age of 60 or more than 60 years old	3.10E-13
The systolic blood pressure between 80-139mmHg	The age of 60 or more than 60 years old	The urinary glucose: Borderline levels	4.22E-13
The habitual smoking: No	The age of 60 or more than 60 years old	The urinary protein:Borderline	7.83E-13
The plasma ALT levels no greater than 40IU/L	The systolic blood pressure between 80-139mmHg	The age of 60 or more than 60 years old	8.18E-13
The plasma HbA1c levels no greater than 6.2%	The age of 60 or more than 60 years old	The plasma triglyceride levels less than 150 mg/dL	9.55E-13
The body mass index between 18.5-24.9	The plasma HbA1c levels no greater than 6.2%	The age of 60 or more than 60 years old	1.04E-12
The systolic blood pressure between 80-139mmHg	The age of 60 or more than 60 years old		1.29E-12
The body mass index between 18.5-24.9	The plasma HDL cholesterol levels more than 40mg/L	The age of 60 or more than 60 years old	1.56E-12

The gender: Male	The age of 60 or more than 60 years old	The fasting plasma glucose levels less than 110mg/dL	2.76E-12
The plasma HDL cholesterol levels more than 40mg/L	The age of 60 or more than 60 years old	The plasma triglyceride levels less than 150 mg/dL	3.33E-12
The plasma LDL cholesterol levels less than 140 mg/dL	The plasma HbA1c levels no greater than 6.2%	The age of 60 or more than 60 years old	5.92E-12
The plasma AST levels no greater than 40IU/L	The use of aspirin	The urinary glucose: Borderline levels	7.66E-12
The plasma AST levels no greater than 40IU/L	The diastolic blood pressure less than 90mmHg	The age of 60 or more than 60 years old	8.59E-12
The plasma HDL cholesterol levels more than 40mg/L	The plasma LDL cholesterol levels less than 140 mg/dL	The age of 60 or more than 60 years old	1.10E-11
The diastolic blood pressure less than 90mmHg	The age of 60 or more than 60 years old	The urinary glucose: Borderline levels	1.36E-11
The body mass index between 18.5-24.9	The age of 60 or more than 60 years old	The urinary protein:Borderline	1.37E-11
The plasma ALT levels no greater than 40IU/L	The use of aspirin	The urinary glucose: Borderline levels	2.01E-11
The use of aspirin	The urinary glucose: Borderline levels		2.08E-11
The plasma AST levels no greater than 40IU/L	The use of aspirin		2.08E-11
The plasma ALT levels no greater than 40IU/L	The diastolic blood pressure less than 90mmHg	The age of 60 or more than 60 years old	2.18E-11
The plasma HDL cholesterol levels more than 40mg/L	The systolic blood pressure between 80-139mmHg	The age of 60 or more than 60 years old	2.27E-11
The habitual smoking: No	The plasma LDL cholesterol levels less than 140 mg/dL	The age of 60 or more than 60 years old	2.60E-11
The age of 60 or more than 60 years old	The plasma triglyceride levels less than 150 mg/dL,	The urinary protein:Borderline	2.86E-11
The gender: Male	The use of aspirin	The urinary glucose: Borderline levels	3.13E-11
The gender: Male	The plasma AST levels no greater than 40IU/L	The use of aspirin	3.13E-11
The plasma HbA1c levels no greater than 6.2%	The diastolic blood pressure less than 90mmHg	The age of 60 or more than 60 years old	4.15E-11
The plasma ALT levels no greater than 40IU/L	The plasma AST levels no greater than 40IU/L	The use of aspirin	4.25E-11
The diastolic blood pressure less than 90mmHg	The age of 60 or more than 60 years old		4.91E-11
The plasma HDL cholesterol levels more than 40mg/L	The plasma AST levels no greater than 40IU/L	The use of aspirin	5.11E-11
The plasma ALT levels no greater than 40IU/L	The use of aspirin		5.11E-11

The plasma HDL cholesterol levels more than 40mg/L	The use of aspirin	The urinary glucose: Borderline levels	5.60E-11
The use of aspirin			5.89E-11
The systolic blood pressure between 80-139mmHg	The age of 60 or more than 60 years old	The plasma γ-GTP levels no greater than 71IU/L	6.92E-11
The plasma ALT levels no greater than 40IU/L	The gender: Male	The use of aspirin	6.97E-11
The gender: Male	The use of aspirin		9.24E-11
The habitual smoking: No	The age of 60 or more than 60 years old	The plasma triglyceride levels less than 150 mg/dL	1.30E-10
The plasma HDL cholesterol levels more than 40mg/L	The plasma HbA1c levels no greater than 6.2%	The use of aspirin	1.44E-10
The plasma HDL cholesterol levels more than 40 mg/dL	The use of aspirin		1.48E-10
The plasma HDL cholesterol levels more than 40mg/L	The diastolic blood pressure less than 90mmHg	The age of 60 or more than 60 years old	1.62E-10
The plasma ALT levels no greater than 40IU/L	The plasma HDL cholesterol levels more than 40 mg/dL	The use of aspirin	1.63E-10
The plasma HbA1c levels no greater than 6.2%	The plasma AST levels no greater than 40IU/L	The use of aspirin	1.76E-10
The age of 60 or more than 60 years old	The plasma triglyceride levels less than 150 mg/dL,	The fasting plasma glucose levels less than 110mg/dL	1.77E-10
The body mass index between 18.5-24.9	The age of 60 or more than 60 years old	The fasting plasma glucose levels less than 110mg/dL	1.96E-10
The systolic blood pressure between 80-139mmHg	The age of 60 or more than 60 years old	The urinary protein:Borderline	2.12E-10
The plasma HDL cholesterol levels more than 40mg/L	The gender: Male	The use of aspirin	2.37E-10
The gender: Male	The age of 60 or more than 60 years old	The plasma triglyceride levels less than 150 mg/dL	2.89E-10
The gender: Male	The age of 60 or more than 60 years old	The urinary protein:Borderline	3.16E-10
The plasma HbA1c levels no greater than 6.2%	The use of aspirin	The urinary glucose: Borderline levels	3.51E-10
The body mass index between 18.5-24.9	The habitual smoking: No	The age of 60 or more than 60 years old	3.84E-10

The gender: Male	The plasma LDL cholesterol levels less than 140 mg/dL	The age of 60 or more than 60 years old	3.99E-10
The systolic blood pressure between 80-139mmHg	The diastolic blood pressure less than 90mmHg	The age of 60 or more than 60 years old	4.33E-10
The plasma HbA1c levels no greater than 6.2%	The use of aspirin		4.67E-10
The habitual smoking: No	The systolic blood pressure between 80-139mmHg	The age of 60 or more than 60 years old	7.16E-10
The plasma ALT levels no greater than 40IU/L	The plasma HbA1c levels no greater than 6.2%	The use of aspirin	7.30E-10
The gender: Male	The use of aspirin	The plasma HbA1c levels no greater than 6.2%	7.60E-10
The body mass index between 18.5-24.9	The gender: Male	The age of 60 or more than 60 years old	8.10E-10
The diastolic blood pressure less than 90mmHg	The age of 60 or more than 60 years old	The plasma γ-GTP levels no greater than 71IU/L	1.01E-09
The systolic blood pressure between 80-139mmHg	The age of 60 or more than 60 years old	The fasting plasma glucose levels less than 110mg/dL	1.36E-09
The plasma LDL cholesterol levels less than 140 mg/dL	The age of 60 or more than 60 years old	The urinary protein:Borderline	3.37E-09
The systolic blood pressure between 80-139mmHg	The age of 60 or more than 60 years old	The plasma triglyceride levels less than 150 mg/dL	6.52E-09
The diastolic blood pressure less than 90mmHg	The age of 60 or more than 60 years old	The urinary protein:Borderline	1.11E-08
The diastolic blood pressure less than 90mmHg	The age of 60 or more than 60 years old	The fasting plasma glucose levels less than 110mg/dL	2.68E-08
The habitual smoking: No	The diastolic blood pressure less than 90mmHg	The age of 60 or more than 60 years old	3.81E-08
The body mass index between 18.5-24.9	The age of 60 or more than 60 years old	The plasma triglyceride levels less than 150 mg/dL	4.04E-08
The plasma LDL cholesterol levels less than 140 mg/dL	The age of 60 or more than 60 years old	The fasting plasma glucose levels less than 110mg/dL	5.73E-08
The plasma ALT levels no greater than 40IU/L	The use of aspirin	The plasma γ-GTP levels no greater than 71IU/L	6.47E-08
The plasma LDL cholesterol levels less than 140 mg/dL	The systolic blood pressure between 80-139mmHg	The age of 60 or more than 60 years old	7.78E-08
The use of aspirin	The urinary glucose: Borderline	The plasma γ-GTP levels no greater than 71IU/L	9.83E-08

The plasma AST levels no greater than 40IU/L	The use of aspirin	The plasma γ-GTP levels no greater than 71IU/L	1.48E-07
The use of aspirin	The plasma γ-GTP levels no greater than 71IU/L		2.20E-07
The habitual smoking: No	The plasma ALT levels no greater than 40IU/L	The use of aspirin	3.48E-07
The gender: MaleThe use of aspirin	The plasma γ-GTP levels no greater than 71IU/L		3.94E-07
The plasma LDL cholesterol levels less than 140 mg/dL	The diastolic blood pressure less than 90mmHg	The age of 60 or more than 60 years old	5.02E-07
The gender: Male	The diastolic blood pressure less than 90mmHg	The age of 60 or more than 60 years old	5.11E-07
The plasma ALT levels no greater than 40IU/L	The use of aspirin	The urinary protein:Borderline	5.68E-07
The habitual smoking: No	The plasma HbA1c levels no greater than 6.2%	The use of aspirin	6.16E-07
The plasma HDL cholesterol levels more than 40 mg/dL	The use of aspirin	The plasma γ-GTP levels no greater than 71IU/L	6.16E-07
The plasma HDL cholesterol levels more than 40 mg/dL	The urinary glucose: Borderline	The use of warfarin	6.29E-07
The plasma HDL cholesterol levels more than 40 mg/dL	The use of warfarin		8.74E-07
The habitual smoking: No	The plasma HDL cholesterol levels more than 40mg/L	The use of aspirin	9.16E-07
The habitual smoking: No	The plasma AST levels no greater than 40IU/L	The use of aspirin	9.90E-07
The habitual smoking: No	The use of aspirin	The urinary glucose: Borderline levels	1.07E-06
The plasma LDL cholesterol levels less than 140 mg/dL	The age of 60 or more than 60 years old	The plasma triglyceride levels less than 150 mg/dL	1.11E-06
The body mass index between 18.5-24.9	The plasma LDL cholesterol levels less than 140 mg/dL	The age of 60 or more than 60 years old	1.28E-06
The body mass index between 18.5-24.9	The diastolic blood pressure less than 90mmHg	The age of 60 or more than 60 years old	1.56E-06
The plasma AST levels no greater than 40IU/L	The use of aspirin	The urinary protein:Borderline	1.57E-06
The gender: Male	The systolic blood pressure between 80-139mmHg	The age of 60 or more than 60 years old	1.57E-06
The plasma AST levels no greater than 40IU/L	The exercise habits more than 30min per day: No	The age of 60 or more than 60 years old	1.67E-06

The use of aspirin	The urinary glucose: Borderline	The urinary protein:Borderline	1.82E-06
The urinary glucose: Borderline	The use of warfarin		1.92E-06
The body mass index between 18.5-24.9	The systolic blood pressure between 80-139mmHg	The age of 60 or more than 60 years old	1.99E-06
The habitual smoking: No	The use of aspirin		2.11E-06
The exercise habits more than 30min per day: No	The age of 60 or more than 60 years old	The urinary glucose: Borderline levels	2.18E-06
The plasma AST levels no greater than 40IU/L	The age of 60 or more than 60 years old	The body weight gain more than 2kg a year: No	2.32E-06
The use of warfarin			3.00E-06
The urinary glucose: Borderline	The age of 60 or more than 60 years old	The body weight gain more than 2kg a year: No	3.11E-06
The use of aspirin	The urinary protein:Borderline		3.52E-06
The plasma HbA1c levels no greater than 6.2%	The use of aspirin	The plasma γ-GTP levels no greater than 71IU/L	3.96E-06
The diastolic blood pressure less than 90mmHg	The age of 60 or more than 60 years old	The plasma triglyceride levels less than 150 mg/dL	4.74E-06
The exercise habits more than 30min per day: No	The age of 60 or more than 60 years old		5.33E-06
The habitual smoking: No	The gender: Male	The use of aspirin	5.55E-06
The plasma LDL cholesterol levels less than 140 mg/dL	The use of aspirin	The urinary glucose: Borderline levels	5.59E-06
The plasma HbA1c levels no greater than 6.2%	The exercise habits more than 30min per day: No	The use of aspirin	5.65E-06
The plasma HbA1c levels no greater than 6.2%	The exercise habits more than 30min per day: No	The age of 60 or more than 60 years old	6.45E-06
The plasma ALT levels no greater than 40IU/L	The exercise habits more than 30min per day: No	The age of 60 or more than 60 years old	6.85E-06
The plasma LDL cholesterol levels less than 140 mg/dL	The plasma AST levels no greater than 40IU/L	The use of aspirin	7.00E-06
The plasma HbA1c levels no greater than 6.2%	The use of aspirin	The urinary protein:Borderline	7.72E-06
The age of 60 or more than 60 years old	The body weight gain more than 2kg a year: No		8.09E-06

The gender: MaleThe exercise habits more than 30min per day: No	The age of 60 or more than 60 years old		8.70E-06
The plasma LDL cholesterol levels between 140-200mg/dL	The age of 60 or more than 60 years old	The fasting plasma glucose levels less than 110mg/dL	1.06E-05
The plasma HDL cholesterol levels more than 40mg/L	The use of aspirin	The urinary protein:Borderline	1.09E-05
The gender: Male	The use of aspirin	The urinary protein:Borderline	1.15E-05
The plasma LDL cholesterol levels less than 140 mg/dL	The use of aspirin		1.26E-05
The habitual smoking: No	The exercise habits more than 30min per day: No	The age of 60 or more than 60 years old	1.42E-05
The plasma HDL cholesterol levels more than 40mg/L	The plasma triglyceride levels less than 150 mg/dL,	The use of warfarin	1.52E-05
The plasma HDL cholesterol levels more than 40mg/L	The exercise habits more than 30min per day: No	The age of 60 or more than 60 years old	1.53E-05
The use of aspirin	The urinary glucose: Borderline	The plasma triglyceride levels less than 150 mg/dL	1.58E-05
The habitual smoking: No	The age of 60 or more than 60 years old	The body weight gain more than 2kg a year: No	1.81E-05
The urinary glucose: Borderline	The plasma triglyceride levels less than 150 mg/dL,	The use of warfarin	1.82E-05
The plasma HDL cholesterol levels more than 40mg/L	The plasma LDL cholesterol levels less than 140 mg/dL	The use of warfarin	1.82E-05
The plasma AST levels no greater than 40IU/L	The use of aspirin	The plasma triglyceride levels less than 150 mg/dL	1.99E-05
The exercise habits more than 30min per day: No	The use of aspirin	The urinary glucose: Borderline levels	2.18E-05
The plasma HDL cholesterol levels more than 40mg/L	The exercise habits more than 30min per day: No	The use of aspirin	2.18E-05
The plasma HbA1c levels no greater than 6.2%	The use of aspirin	The fasting plasma glucose levels less than 110mg/dL	2.18E-05

The plasma ALT levels no greater than 40IU/L	The age of 60 or more than 60 years old	The body weight gain more than 2kg a year: No	2.28E-05
The exercise habits more than 30min per day: No	The age of 60 or more than 60 years old	The plasma γ-GTP levels no greater than 71IU/L	2.47E-05
The plasma triglyceride levels less than 150 mg/dL,	The use of warfarin		2.58E-05
The plasma ALT levels no greater than 40IU/L	The plasma LDL cholesterol levels less than 140 mg/dL	The use of aspirin	2.68E-05
The plasma AST levels no greater than 40IU/L	The exercise habits more than 30min per day: No	The use of aspirin	3.31E-05
The plasma HDL cholesterol levels more than 40mg/L	The use of aspirin	The fasting plasma glucose levels less than 110mg/dL	3.31E-05
The use of aspirin	The plasma triglyceride levels less than 150 mg/dL		3.35E-05
The gender: Male	The systolic blood pressure between 80-139mmHg	The use of aspirin	3.39E-05
The plasma LDL cholesterol levels less than 140 mg/dL	The urinary glucose: Borderline	The use of warfarin	3.61E-05
The plasma ALT levels no greater than 40IU/L	The use of aspirin	The fasting plasma glucose levels less than 110mg/dL	3.67E-05
The plasma HbA1c levels no greater than 6.2%	The age of 60 or more than 60 years old	The body weight gain more than 2kg a year: No	3.70E-05
The plasma HbA1c levels no greater than 6.2%	The plasma LDL cholesterol levels between 140-200mg/dL	The age of 60 or more than 60 years old	3.82E-05
The plasma HDL cholesterol levels more than 40mg/L	The plasma LDL cholesterol levels less than 140 mg/dL	The use of aspirin	3.87E-05
The plasma AST levels no greater than 40IU/L	The use of aspirin	The fasting plasma glucose levels less than 110mg/dL	4.49E-05
The gender: Male	The use of aspirin	The age of 60 or more than 60 years old	4.96E-05
The drinking alcohol at present: Not everyday but sometime	The gender: Male	The use of aspirin	5.01E-05
The plasma LDL cholesterol levels less than 140 mg/dL	The use of warfarin		5.01E-05
The exercise habits more than 30min per day: No	The use of aspirin		5.48E-05

The systolic blood pressure between 90-110mmHg	The plasma γ-GTP levels no greater than 71IU/L		5.50E-05
The systolic blood pressure between 90-110mmHg	The urinary glucose: Borderline levels		5.60E-05
The body weight gain more than 2kg a year: No	The plasma ALT levels no greater than 40IU/L	The age of 60 or more than 60 years old	5.63E-05
The plasma AST levels no greater than 40IU/L	The systolic blood pressure between 90-110mmHg	The plasma γ-GTP levels no greater than 71IU/L	5.72E-05
The plasma HDL cholesterol levels more than 40mg/L	The gender: Male	The use of warfarin	5.88E-05
The gender: Male	The use of aspirin	The plasma LDL cholesterol levels less than 140 mg/dL	6.05E-05
The plasma triglyceride levels less than 150 mg/dL,	The use of warfarin	The plasma γ-GTP levels no greater than 71IU/L	6.55E-05
The diastolic blood pressure between 90-110mmHg			7.21E-05
The use of aspirin	The fasting plasma glucose levels less than 110mg/dL		7.33E-05
The use of aspirin	The fasting plasma glucose levels less than 110mg/dL,	The urinary glucose: Borderline levels	7.33E-05
The systolic blood pressure between 90-110mmHg	The urinary glucose: Borderline	The plasma γ-GTP levels no greater than 71IU/L	7.46E-05
The plasma ALT levels no greater than 40IU/L	The use of aspirin	The plasma triglyceride levels less than 150 mg/dL	7.62E-05
The plasma AST levels no greater than 40IU/L	The diastolic blood pressure between 90-110mmHg		8.07E-05
The plasma ALT levels no greater than 40IU/L	The age of 60 or more than 60 years old	The body weight gain more than 2kg a year: Yes	8.23E-05
The age of 60 or more than 60 years old	The body weight gain more than 2kg a year: No,	The urinary protein:Borderline	8.68E-05
The plasma AST levels no greater than 40IU/L	The systolic blood pressure between 90-110mmHg	The urinary glucose: Borderline levels	8.72E-05
The age of 60 or more than 60 years old	The body weight gain more than 2kg a year: No,	The plasma γ-GTP levels no greater than 71IU/L	8.82E-05
The plasma triglyceride levels less than 150 mg/dL,	The use of rosuvastatin	The use of warfarin	9.05E-05
The gender: Male	The use of aspirin	The plasma triglyceride levels less than 150 mg/dL	9.61E-05

The body weight gain more than 2kg a year: No	The age of 60 or more than 60 years old	The plasma γ-GTP levels no greater than 71IU/L	0.000104
The plasma HbA1c levels no greater than 6.2%	The systolic blood pressure between 90-110mmHg	The urinary glucose: Borderline levels	0.00010401
The drinking alcohol at present: Not everyday but sometime	The use of aspirin	The plasma HDL cholesterol levels more than 40 mg/dL	0.00010854
The plasma HDL cholesterol levels more than 40mg/L	The plasma HbA1c levels no greater than 6.2%	The use of warfarin	0.00010854
The plasma ALT levels no greater than 40IU/L	The systolic blood pressure between 90-110mmHg	The plasma γ-GTP levels no greater than 71IU/L	0.00010949
The diastolic blood pressure less than 90mmHg	The gender: Male	The use of aspirin	0.00011333
The gender: Male	The urinary glucose: Borderline	The use of warfarin	0.00012579
The plasma AST levels no greater than 40IU/L	The plasma LDL cholesterol levels between 140-200mg/dL	The age of 60 or more than 60 years old	0.00013263
The plasma HbA1c levels no greater than 6.2%	The diastolic blood pressure between 90-110mmHg		0.00013896
The plasma ALT levels no greater than 40IU/L	The use of aspirin	The age of 60 or more than 60 years old	0.00014099
The plasma HbA1c levels no greater than 6.2%	The systolic blood pressure between 90-110mmHg	The plasma γ-GTP levels no greater than 71IU/L	0.0001419
The plasma HDL cholesterol levels more than 40mg/L	The plasma AST levels no greater than 40IU/L	The use of warfarin	0.00014546
The plasma LDL cholesterol levels between 140-200mg/dL	The age of 60 or more than 60 years old	The urinary glucose: Borderline levels	0.00014841
The plasma HbA1c levels no greater than 6.2%	The age of 60 or more than 60 years old	The plasma triglyceride levels between 150-300mg/dL	0.00014912
The plasma HbA1c levels no greater than 6.2%	The age of 60 or more than 60 years old	The body weight gain more than 2kg a year: Yes	0.0001507
The body weight gain more than 2kg a year: No	The plasma AST levels no greater than 40IU/L	The age of 60 or more than 60 years old	0.00015144
The plasma ALT levels no greater than 40IU/L	The plasma LDL cholesterol levels between 140-200mg/dL	The age of 60 or more than 60 years old	0.00015496
The plasma ALT levels no greater than 40IU/L	The drinking alcohol at present: None	The age of 60 or more than 60 years old	0.00015961
The plasma ALT levels no greater than 40IU/L	The plasma AST levels no greater than 40IU/L	The diastolic blood pressure between 90-110mmHg	0.00015972

The plasma HDL cholesterol levels more than 40mg/L	The age of 60 or more than 60 years old	The body weight gain more than 2kg a year: Yes	0.00016353
The plasma ALT levels no greater than 40IU/L	The exercise habits more than 30min per day: No	The use of aspirin	0.00016688
The plasma LDL cholesterol levels less than 140 mg/dL	The use of aspirin	The plasma HbA1c levels no greater than 6.2%	0.00016963
The plasma HDL cholesterol levels more than 40mg/L	The use of aspirin	The plasma triglyceride levels less than 150 mg/dL	0.00017114
The body weight gain more than 2kg a year: No	The age of 60 or more than 60 years old	The urinary glucose: Borderline levels	0.00017776
The plasma AST levels no greater than 40IU/L	The age of 60 or more than 60 years old	The body weight gain more than 2kg a year: Yes	0.00018691
The plasma ALT levels no greater than 40IU/L	The exercise habits more than 30min per day: No	The age of 60 or more than 60 years old	0.00018808
The drinking alcohol at present: Not everyday but sometime	The use of aspirin	The urinary glucose: Borderline levels	0.00019328
The gender: Male	The use of warfarin		0.00019328
The plasma LDL cholesterol levels less than 140 mg/dL	The use of rosuvastatin	The use of warfarin	0.0001974
The age of 60 or more than 60 years old	The urinary glucose: Borderline	The body weight gain more than 2kg a year: Yes	0.0002042
The plasma ALT levels no greater than 40IU/L	The diastolic blood pressure between 90-110mmHg		0.00020465
The body weight gain more than 2kg a year: No	The age of 60 or more than 60 years old	The fasting plasma glucose levels less than 110mg/dL	0.00022578
The urinary protein:Borderline	The use of aspirin	The plasma γ-GTP levels no greater than 71IU/L	0.00022688
The plasma HDL cholesterol levels more than 40mg/L	The use of warfarin	The plasma γ-GTP levels no greater than 71IU/L	0.0002322
The plasma LDL cholesterol levels between 140-200mg/dL	The age of 60 or more than 60 years old		0.00024695
The plasma HbA1c levels no greater than 6.2%	The urinary glucose: Borderline	The use of warfarin	0.00025476
The plasma AST levels no greater than 40IU/L	The use of aspirin	The age of 60 or more than 60 years old	0.00026173
The gender: Male	The plasma LDL cholesterol levels less than 140 mg/dL	The use of warfarin	0.00027443

The plasma ALT levels no greater than 40IU/L	The systolic blood pressure between 90-110mmHg	The urinary glucose: Borderline levels	0.00027487
The use of aspirin	The age of 60 or more than 60 years old	The urinary glucose: Borderline levels	0.00028516
The drinking alcohol at present: Not everyday but sometime	The plasma AST levels no greater than 40IU/L	The use of aspirin	0.00029163
The plasma AST levels no greater than 40IU/L	The urinary glucose: Borderline	The use of warfarin	0.00029163
The plasma HbA1c levels no greater than 6.2%	The use of warfarin		0.00029163
The systolic blood pressure between 80-139mmHg	The use of aspirin	The urinary glucose: Borderline levels	0.00029194
The drinking alcohol at present: None	The age of 60 or more than 60 years old	The plasma γ-GTP levels no greater than 71IU/L	0.00030075
The plasma AST levels no greater than 40IU/L	The systolic blood pressure between 80-139mmHg	The use of aspirin	0.00031487
The gender: Male	The use of aspirin	The fasting plasma glucose levels less than 110mg/dL	0.00032167
The drinking alcohol at present: Not everyday but sometime	The use of aspirin		0.00033322
The age of 60 or more than 60 years old	The body weight gain more than 2kg a year: Yes		0.00034354
The plasma HDL cholesterol levels more than 40mg/L	The age of 60 or more than 60 years old	The body weight gain more than 2kg a year: No	0.00034545
The plasma γ-GTP levels no greater than 71IU/L,	The exercise habits more than 30min per day: No	The age of 60 or more than 60 years old	0.00034599
The plasma HbA1c levels no greater than 6.2%	The plasma AST levels no greater than 40IU/L	The diastolic blood pressure between 90-110mmHg	0.00035136
The plasma AST levels no greater than 40IU/L	The plasma triglyceride levels less than 150 mg/dL,	The use of warfarin	0.00037986
The plasma AST levels no greater than 40IU/L	The use of rosuvastatin	The use of warfarin	0.00039148
The body weight gain more than 2kg a year: No	The age of 60 or more than 60 years old		0.00039735
The use of aspirin	The age of 60 or more than 60 years old		0.00039927
The plasma AST levels no greater than 40IU/L	The drinking alcohol at present: None	The age of 60 or more than 60 years old	0.00041892

The plasma AST levels no greater than 40IU/L	The use of warfarin		0.00043269
The urinary glucose: Borderline	The use of warfarin	The plasma γ-GTP levels no greater than 71IU/L	0.00044498
The drinking alcohol at present: None	The age of 60 or more than 60 years old	The urinary glucose: Borderline levels	0.00046846
The plasma AST levels no greater than 40IU/L	The exercise habits more than 30min per day: No	The age of 60 or more than 60 years old	0.00050079
The drinking alcohol at present: Not everyday but sometime	The plasma HbA1c levels no greater than 6.2%	The use of aspirin	0.00051984
The use of aspirin	The plasma triglyceride levels less than 150 mg/dL,	The plasma γ-GTP levels no greater than 71IU/L	0.00052761
The plasma ALT levels no greater than 40IU/L	The body mass index between 25.0-30.0	The age of 60 or more than 60 years old	0.0005374
The exercise habits more than 30min per day: No	The urinary glucose: Borderline	The age of 60 or more than 60 years old	0.00055944
The systolic blood pressure between 80-139mmHg	The use of aspirin		0.00056697
The plasma AST levels no greater than 40IU/L	The age of 60 or more than 60 years old	The plasma triglyceride levels between 150-300mg/dL	0.00057972
The gender: Male	The exercise habits more than 30min per day: No	The use of aspirin	0.00061621
The drinking alcohol at present: None	The age of 60 or more than 60 years old	The fasting plasma glucose levels less than 110mg/dL	0.00064971
The body mass index between 25.0-30.0	The plasma AST levels no greater than 40IU/L	The age of 60 or more than 60 years old	0.00068455
The use of warfarin	The plasma γ-GTP levels no greater than 71IU/L		0.00070387
The urinary glucose: Borderline	The use of rosuvastatin	The use of warfarin	0.00072094
The plasma ALT levels no greater than 40IU/L	The age of 60 or more than 60 years old	The plasma triglyceride levels between 150-300mg/dL	0.00073541
The diastolic blood pressure less than 90mmHg	The use of aspirin	The plasma HDL cholesterol levels more than 40 mg/dL	0.0007528
The plasma HbA1c levels no greater than 6.2%	The use of aspirin	The plasma triglyceride levels less than 150 mg/dL	0.00076082
The fasting plasma glucose levels less than 110mg/dL,	The exercise habits more than 30min per day: No	The age of 60 or more than 60 years old	0.00077102

The plasma LDL cholesterol levels between 140-200mg/dL	The age of 60 or more than 60 years old	The plasma γ-GTP levels no greater than 71IU/L	0.00078538
The diastolic blood pressure less than 90mmHg	The plasma HbA1c levels no greater than 6.2%	The use of aspirin	0.0008226
The drinking alcohol at present: None	The exercise habits more than 30min per day: No	The age of 60 or more than 60 years old	0.00082912
The body weight gain more than 2kg a year: No	The drinking alcohol at present: None	The age of 60 or more than 60 years old	0.00084724
The plasma LDL cholesterol levels less than 140 mg/dL	The use of aspirin	The plasma γ-GTP levels no greater than 71IU/L	0.00086527
The age of 60 or more than 60 years old	The urinary glucose: Borderline	The plasma triglyceride levels between 150-300mg/dL	0.00088996
The body weight gain more than 2kg a year: No	The plasma HbA1c levels no greater than 6.2%	The age of 60 or more than 60 years old	0.00092341
The diastolic blood pressure less than 90mmHg	The plasma AST levels no greater than 40IU/L	The use of aspirin	0.00092709
The plasma γ-GTP levels no greater than 71IU/L	The drinking alcohol at present: Not everyday but sometime	The use of aspirin	0.00093624
The plasma HDL cholesterol levels more than 40mg/L	The plasma LDL cholesterol levels between 140-200mg/dL	The age of 60 or more than 60 years old	0.000985
The diastolic blood pressure less than 90mmHg	The use of aspirin	The urinary glucose: Borderline levels	0.00099292
The drinking alcohol at present: None	The age of 60 or more than 60 years old		0.0010281
The systolic blood pressure between 80-139mmHg	The age of 60 or more than 60 years old	The body weight gain more than 2kg a year: No	0.0010324
The body weight gain more than 2kg a year: No	The exercise habits more than 30min per day: No	The age of 60 or more than 60 years old	0.001073
The gender: Male	The age of 60 or more than 60 years old	The body weight gain more than 2kg a year: No	0.0010802
The plasma ALT levels no greater than 40IU/L	The plasma HDL cholesterol levels more than 40mg/L	The use of warfarin	0.0010886
The exercise habits more than 30min per day: No	The age of 60 or more than 60 years old		0.001219
The use of rosuvastatin	The use of warfarin		0.001251
The plasma HDL cholesterol levels more than 40mg/L	The use of rosuvastatin	The use of warfarin	0.001251

The plasma HbA1c levels no greater than 6.2%	The use of aspirin	The age of 60 or more than 60 years old	0.0012526
The plasma γ-GTP levels no greater than 71IU/L,	The age of 60 or more than 60 years old	The body weight gain more than 2kg a year: Yes	0.001388
The drinking alcohol at present: None	The plasma HbA1c levels no greater than 6.2%	The age of 60 or more than 60 years old	0.0013982
The plasma ALT levels no greater than 40IU/L	The drinking alcohol at present: Not everyday but sometime	The use of aspirin	0.0014393
The habitual smoking: No	The use of aspirin	The plasma γ-GTP levels no greater than 71IU/L	0.0014863
The gender: Male	The systolic blood pressure between 90-110mmHg	The urinary glucose: Borderline levels	0.0015032
The exercise habits more than 30min per day: No	The age of 60 or more than 60 years old	The urinary protein:Borderline	0.001505
The plasma ALT levels no greater than 40IU/L	The systolic blood pressure between 80-139mmHg	The use of aspirin	0.0015075
The exercise habits more than 30min per day: No	The use of aspirin	The urinary protein:Borderline	0.0015206
The body mass index between 25.0-30.0	The age of 60 or more than 60 years old	The urinary glucose: Borderline levels	0.0015328
The gender: Male	The systolic blood pressure between 90-110mmHg	The plasma γ-GTP levels no greater than 71IU/L	0.0016052
The habitual smoking: No	The plasma HDL cholesterol levels more than 40mg/L	The use of warfarin	0.0016496
The plasma ALT levels no greater than 40IU/L	The urinary glucose: Borderline	The use of rosuvastatin	0.0016524
The drinking alcohol at present: None	The plasma AST levels no greater than 40IU/L	The age of 60 or more than 60 years old	0.0016528
The body weight gain more than 2kg a year: No	The body mass index between 18.5-24.9	The age of 60 or more than 60 years old	0.0017071
The plasma LDL cholesterol levels less than 140 mg/dL	The plasma triglyceride levels less than 150 mg/dL,	The use of warfarin	0.0017671
The plasma HDL cholesterol levels more than 40mg/L	The use of aspirin	The body weight gain more than 2kg a year: No	0.0018013
The gender: Male	The diastolic blood pressure between 90-110mmHg		0.001802
The diastolic blood pressure less than 90mmHg	The use of aspirin		0.0018091

The use of aspirin	The fasting plasma glucose levels less than 110mg/dL,	The plasma γ-GTP levels no greater than 71IU/L	0.0018392
The plasma ALT levels no greater than 40IU/L	The plasma HDL cholesterol levels more than 40mg/L	The use of rosuvastatin	0.0018805
The plasma ALT levels no greater than 40IU/L	The urinary glucose: Borderline	The use of warfarin	0.0018867
The drinking alcohol at present: None	The age of 60 or more than 60 years old	The urinary glucose: Borderline levels	0.0019
The use of aspirin	The urinary glucose: Borderline	The body weight gain more than 2kg a year: No	0.0019547
The plasma HDL cholesterol levels more than 40mg/L	The use of aspirin	The age of 60 or more than 60 years old	0.0019547
The plasma ALT levels no greater than 40IU/L	The plasma HbA1c levels no greater than 6.2%	The diastolic blood pressure between 90-110mmHg	0.0020004
The plasma HDL cholesterol levels more than 40mg/L	The systolic blood pressure between 80-139mmHg	The use of aspirin	0.0020166
The gender: Male	The plasma triglyceride levels less than 150 mg/dL,	The use of warfarin	0.0020871
The plasma ALT levels no greater than 40IU/L	The plasma AST levels no greater than 40IU/L	The use of warfarin	0.0021535
The age of 60 or more than 60 years old	The plasma triglyceride levels between 150-300mg/dL		0.0021579
The habitual smoking: No	The use of aspirin	The fasting plasma glucose levels less than 110mg/dL	0.0022166
The plasma HDL cholesterol levels more than 40mg/L	The systolic blood pressure between 90-110mmHg	The urinary glucose: Borderline levels	0.0022343
The gender: Male	The plasma AST levels no greater than 40IU/L	The diastolic blood pressure between 90-110mmHg	0.0022637
The plasma HDL cholesterol levels more than 40mg/L	The plasma HbA1c levels no greater than 6.2%	The diastolic blood pressure between 90-110mmHg	0.0022953
The plasma ALT levels no greater than 40IU/L	The plasma triglyceride levels less than 150 mg/dL,	The use of warfarin	0.0024569
The habitual smoking: No	The use of aspirin	The urinary protein:Borderline	0.0024923
The plasma HDL cholesterol levels more than 40mg/L	The diastolic blood pressure between 90-110mmHg		0.0024944
The diastolic blood pressure less than 90mmHg	The systolic blood pressure between 80-139mmHg	The use of aspirin	0.0024961

The drinking alcohol at present: None	The age of 60 or more than 60 years old		0.0025028
The plasma HbA1c levels no greater than 6.2%	The drinking alcohol at present: None	The age of 60 or more than 60 years old	0.0025028
The plasma LDL cholesterol levels between 140-200mg/dL	The age of 60 or more than 60 years old	The urinary protein:Borderline	0.0025902
The plasma HbA1c levels no greater than 6.2%	The use of aspirin	The body weight gain more than 2kg a year: No	0.0026624
The exercise habits more than 30min per day: No	The age of 60 or more than 60 years old	The fasting plasma glucose levels less than 110mg/dL	0.0026795
The body mass index between 25.0-30.0	The age of 60 or more than 60 years old		0.0027545
The plasma ALT levels no greater than 40IU/L	The use of warfarin		0.0027892
The plasma AST levels no greater than 40IU/L	The use of aspirin	The body weight gain more than 2kg a year: No	0.0029144
The plasma HbA1c levels no greater than 6.2%	The exercise habits more than 30min per day: No	The age of 60 or more than 60 years old	0.0029334
The habitual smoking: No	The urinary glucose: Borderline	The use of warfarin	0.0031655
The body mass index between 25.0-30.0	The plasma HbA1c levels no greater than 6.2%	The age of 60 or more than 60 years old	0.0032639
The plasma LDL cholesterol levels less than 140 mg/dL	The use of aspirin	The plasma triglyceride levels less than 150 mg/dL	0.0032966
The plasma ALT levels no greater than 40IU/L	The plasma AST levels no greater than 40IU/L	The use of rosuvastatin	0.0033849
The plasma HbA1c levels no greater than 6.2%	The systolic blood pressure between 80-139mmHg	The use of aspirin	0.0034051
The plasma LDL cholesterol levels less than 140 mg/dL	The age of 60 or more than 60 years old	The body weight gain more than 2kg a year: No	0.0034738
The plasma ALT levels no greater than 40IU/L	The diastolic blood pressure less than 90mmHg	The use of aspirin	0.0035301
The plasma AST levels no greater than 40IU/L	The exercise habits more than 30min per day: Yes	The age of 60 or more than 60 years old	0.0037603
The fasting plasma glucose levels less than 110mg/dL,	The age of 60 or more than 60 years old	The body weight gain more than 2kg a year: Yes	0.0038046
The plasma ALT levels no greater than 40IU/L	The use of rosuvastatin		0.0038268

The plasma HbA1c levels no greater than 6.2%	The plasma triglyceride levels less than 150 mg/dL,	The use of warfarin	0.0039326
The use of aspirin	The body weight gain more than 2kg a year: No		0.0039693
The plasma HDL cholesterol levels more than 40mg/L	The systolic blood pressure between 90-110mmHg	The plasma γ-GTP levels no greater than 71IU/L	0.0040077
The plasma HDL cholesterol levels more than 40mg/L	The plasma AST levels no greater than 40IU/L	The diastolic blood pressure between 90-110mmHg	0.0041469
The gender: Male	The use of aspirin	The body weight gain more than 2kg a year: No	0.0041496
The body weight gain more than 2kg a year: No	The plasma HDL cholesterol levels more than 40mg/L	The age of 60 or more than 60 years old	0.004304
The body mass index between 25.0-30.0	The use of aspirin	The drinking alcohol at present: not everyday but sometime	0.0043203
The systolic blood pressure between 90-110mmHg	The urinary glucose: Borderline	The urinary protein:Borderline	0.004372
The gender: Male	The plasma HbA1c levels no greater than 6.2%	The diastolic blood pressure between 90-110mmHg	0.0044032
The exercise habits more than 30min per day: Yes	The age of 60 or more than 60 years old	The urinary glucose: Borderline levels	0.0045222
The habitual smoking: No	The use of warfarin		0.0045778
The plasma HDL cholesterol levels more than 40mg/L	The plasma AST levels no greater than 40IU/L	The use of rosuvastatin	0.0047017
The urinary protein:Borderline	The systolic blood pressure between 90-110mmHg	The plasma γ-GTP levels no greater than 71IU/L	0.0048631
The plasma γ-GTP levels no greater than 71IU/L,	The age of 60 or more than 60 years old	The plasma triglyceride levels between 150-300mg/dL	0.0050815
The body mass index between 18.5-24.9	The drinking alcohol at present: None	The age of 60 or more than 60 years old	0.0052132
The plasma AST levels no greater than 40IU/L	The urinary glucose: Borderline	The use of rosuvastatin	0.0052621
The plasma HDL cholesterol levels more than 40mg/L	The fasting plasma glucose levels less than 110mg/dL,	The use of warfarin	0.0055354
The drinking alcohol at present: None	The age of 60 or more than 60 years old	The fasting plasma glucose levels less than 110mg/dL	0.0055726
The gender: Male	The systolic blood pressure more than 140mmHg,	The urinary glucose: Borderline levels	0.0058531

The plasma ALT levels no greater than 40IU/L	The use of rosuvastatin	The plasma γ-GTP levels no greater than 71IU/L	0.005917
The body mass index between 18.5-24.9	The exercise habits more than 30min per day: No	The age of 60 or more than 60 years old	0.0060168
The plasma AST levels no greater than 40IU/L	The exercise habits more than 30min per day: No	The diastolic blood pressure between 90-110mmHg	0.0060804
The plasma LDL cholesterol levels less than 140 mg/dL	The plasma HbA1c levels no greater than 6.2%	The use of warfarin	0.0061329
The plasma HbA1c levels no greater than 6.2%	The systolic blood pressure between 90-110mmHg	The urinary protein:Borderline	0.0061625
The plasma ALT levels no greater than 40IU/L	The gender: Male	The diastolic blood pressure between 90-110mmHg	0.0062401
The plasma triglyceride levels less than 150 mg/dL,	The age of 60 or more than 60 years old	The body weight gain more than 2kg a year: No	0.0066134
The habitual smoking: No	The fasting plasma glucose levels less than 110mg/dL,	The use of warfarin	0.0066742
The fasting plasma glucose levels less than 110mg/dL,	The age of 60 or more than 60 years old	The plasma triglyceride levels between 150-300mg/dL	0.0068188
The body weight gain more than 2kg a year: No	The gender: Male	The plasma HbA1c levels no greater than 6.2%	0.0068785
The use of aspirin	The age of 60 or more than 60 years old	The plasma γ-GTP levels no greater than 71IU/L	0.006893
The plasma ALT levels no greater than 40IU/L	The use of warfarin	The plasma γ-GTP levels no greater than 71IU/L	0.0070743
The plasma ALT levels no greater than 40IU/L	The exercise habits more than 30min per day: Yes	The age of 60 or more than 60 years old	0.0074019
The habitual smoking: No	The age of 60 or more than 60 years old	The plasma triglyceride levels between 150-300mg/dL	0.0075566
The exercise habits more than 30min per day: Yes	The age of 60 or more than 60 years old		0.0079207
The plasma ALT levels no greater than 40IU/L	The body mass index between 25.0-30.0	The use of aspirin	0.0081397
The age of 60 or more than 60 years old	The use of aspirin	The urinary glucose: Borderline levels	0.0083792
The age of 60 or more than 60 years old	The use of aspirin	The plasma HDL cholesterol levels more than 40 mg/dL	0.0083792
The exercise habits more than 30min per day: No	The diastolic blood pressure between 90-110mmHg		0.0088149

The plasma HDL cholesterol levels more than 40mg/L	The urinary glucose: Borderline	The use of rosuvastatin	0.0091149
The plasma AST levels no greater than 40IU/L	The use of warfarin	The plasma γ-GTP levels no greater than 71IU/L	0.0093428
The plasma HDL cholesterol levels more than 40mg/L	The diastolic blood pressure less than 90mmHg	The use of warfarin	0.0093428
The plasma LDL cholesterol levels less than 140 mg/dL	The plasma AST levels no greater than 40IU/L	The use of warfarin	0.0093428
The body weight gain more than 2kg a year: No	The gender: Male	The urinary glucose: Borderline levels	0.0095166
The fasting plasma glucose levels less than 110mg/dL,	The use of warfarin		0.0095692
The urinary glucose: Borderline	The fasting plasma glucose levels less than 110mg/dL,	The use of warfarin	0.0095692
The habitual smoking: No	The body mass index between 25.0-30.0	The age of 60 or more than 60 years old	0.0097856
The age of 60 or more than 60 years old	The plasma AST levels no greater than 40IU/L	The use of aspirin	0.010094
The exercise habits more than 30min per day: No	The systolic blood pressure between 90-110mmHg	The urinary glucose: Borderline levels	0.010117
The plasma ALT levels no greater than 40IU/L	The drinking alcohol at present: None	The age of 60 or more than 60 years old	0.010197
The gender: Male	The systolic blood pressure more than 140mmHg		0.010312
The plasma HDL cholesterol levels more than 40mg/L	The drinking alcohol at present: None	The age of 60 or more than 60 years old	0.010426
The habitual smoking: No	The plasma ALT levels no greater than 40IU/L	The use of rosuvastatin	0.010697
The drinking alcohol at present: Not everyday but sometime	The use of aspirin	The plasma triglyceride levels between 150-300mg/dL	0.010837
The use of aspirin	The urinary glucose: Borderline	The use of warfarin	0.010837
The habitual smoking: No	The exercise habits more than 30min per day: No	The use of aspirin	0.011063
The plasma HDL cholesterol levels more than 40mg/L	The drinking alcohol at present: None	The age of 60 or more than 60 years old	0.011295
The body mass index between 25.0-30.0	The plasma LDL cholesterol levels less than 140 mg/dL	The age of 60 or more than 60 years old	0.011424

The systolic blood pressure between 90-110mmHg	The urinary protein:Borderline		0.011614
The drinking alcohol at present: None	The age of 60 or more than 60 years old	The plasma γ-GTP levels no greater than 71IU/L	0.011649
The body weight gain more than 2kg a year: No	The gender: Male	The age of 60 or more than 60 years old	0.011899
The plasma ALT levels no greater than 40IU/L	The exercise habits more than 30min per day: No	The diastolic blood pressure between 90-110mmHg	0.012097
The plasma AST levels no greater than 40IU/L	The use of rosuvastatin		0.01211
The gender: Male	The diastolic blood pressure less than 90mmHg	The use of warfarin	0.012223
The habitual smoking: No	The plasma HbA1c levels no greater than 6.2%	The diastolic blood pressure between 90-110mmHg	0.012239
The plasma HDL cholesterol levels more than 40mg/L	The exercise habits more than 30min per day: No	The age of 60 or more than 60 years old	0.01275
The plasma ALT levels no greater than 40IU/L	The use of aspirin	The plasma triglyceride levels between 150-300mg/dL	0.013488
The plasma HDL cholesterol levels more than 40mg/L	The use of warfarin	The urinary protein:Borderline	0.013934
The body weight gain more than 2kg a year: No	The gender: Male	The plasma γ-GTP levels no greater than 71IU/L	0.014114
The diastolic blood pressure less than 90mmHg	The age of 60 or more than 60 years old	The body weight gain more than 2kg a year: No	0.01441
The exercise habits more than 30min per day: Yes	The age of 60 or more than 60 years old	The urinary protein:Borderline	0.014732
The chest XP: Normal	The plasma LDL cholesterol levels less than 140 mg/dL	The use of warfarin	0.014934
The plasma LDL cholesterol levels less than 140 mg/dL	VDT: Need for the observation	The use of warfarin	0.014934
The plasma ALT levels no greater than 40IU/L	The use of aspirin	The body weight gain more than 2kg a year: No	0.015206
The age of 60 or more than 60 years old	The body weight gain more than 2kg a year: No,	The fasting plasma glucose levels less than 110mg/dL	0.015304
The plasma HDL cholesterol levels more than 40mg/L	The use of aspirin	The use of warfarin	0.015351
The plasma LDL cholesterol levels less than 140 mg/dL	The use of aspirin	The use of warfarin	0.015351

The habitual smoking: No	The diastolic blood pressure less than 90mmHg	The use of aspirin	0.01536
The plasma HbA1c levels no greater than 6.2%	The use of aspirin	The plasma triglyceride levels between 150-300mg/dL	0.015918
The plasma HDL cholesterol levels more than 40mg/L	The use of rosuvastatin		0.016552
The plasma HbA1c levels no greater than 6.2%	The exercise habits more than 30min per day: No	The diastolic blood pressure between 90-110mmHg	0.016662
The plasma LDL cholesterol levels between 140-200mg/dL	The age of 60 or more than 60 years old	The plasma triglyceride levels less than 150 mg/dL	0.016891
The age of 60 or more than 60 years old	The use of aspirin		0.017266
The habitual smoking: No	The drinking alcohol at present: Not everyday but sometime	The use of aspirin	0.017994
The diastolic blood pressure less than 90mmHg	The urinary glucose: Borderline	The use of warfarin	0.017994
The gender: Male	The plasma HbA1c levels no greater than 6.2%	The use of warfarin	0.017994
The body mass index between 18.5-24.9	The plasma LDL cholesterol levels between 140-200mg/dL	The age of 60 or more than 60 years old	0.018133
The plasma HbA1c levels no greater than 6.2%	The exercise habits more than 30min per day: Yes	The age of 60 or more than 60 years old	0.018545
The drinking alcohol at present: Not everyday but sometime	The exercise habits more than 30min per day: No	The use of aspirin	0.018717
The body weight gain more than 2kg a year: No	The gender: Male		0.019197
The plasma HDL cholesterol levels more than 40mg/L	The age of 60 or more than 60 years old	The plasma triglyceride levels between 150-300mg/dL	0.019453
The plasma ALT levels no greater than 40IU/L	The plasma HDL cholesterol levels more than 40mg/L	The diastolic blood pressure between 90-110mmHg	0.01974
The gender: Male	The plasma LDL cholesterol levels between 140-200mg/dL	The age of 60 or more than 60 years old	0.020017
The urinary glucose: Borderline	The use of warfarin	The urinary protein:Borderline	0.020385
The habitual smoking: No	The plasma ALT levels no greater than 40IU/L	The use of warfarin	0.020385
The urinary glucose: Borderline	The use of rosuvastatin		0.021001

The use of aspirin	The use of warfarin		0.021312
The plasma AST levels no greater than 40IU/L	The use of aspirin	The use of warfarin	0.021312
The plasma HDL cholesterol levels more than 40mg/L	The use of aspirin	The plasma triglyceride levels between 150-300mg/dL	0.021929
The plasma LDL cholesterol levels less than 140 mg/dL	The use of warfarin	The plasma γ-GTP levels no greater than 71IU/L	0.021929
The systolic blood pressure more than 140mmHg,	The urinary glucose: Borderline levels		0.022121
The use of aspirin	The fasting plasma glucose levels less than 110mg/dL,	The urinary protein:Borderline	0.022324
The exercise habits more than 30min per day: No	The age of 60 or more than 60 years old	The body weight gain more than 2kg a year: Yes	0.024359
The habitual smoking: No	The plasma LDL cholesterol levels between 140-200mg/dL	The age of 60 or more than 60 years old	0.025413
The exercise habits more than 30min per day: No	The use of aspirin	The fasting plasma glucose levels less than 110mg/dL	0.025605
The habitual smoking: No	The age of 60 or more than 60 years old	The body weight gain more than 2kg a year: Yes	0.025629
The gender: Male	The plasma HbA1c levels no greater than 6.2%	The exercise habits more than 30min per day: No	0.025717
The use of warfarin	The urinary protein:Borderline		0.026013
The diastolic blood pressure less than 90mmHg	The use of warfarin		0.026013
The gender: Male	The plasma AST levels no greater than 40IU/L	The use of warfarin	0.026013
The gender: Male	The drinking alcohol at present: None	The plasma HbA1c levels no greater than 6.2%	0.026368
The plasma AST levels no greater than 40IU/L	The systolic blood pressure between 90-110mmHg	The urinary protein:Borderline	0.026804
The gender: Male	The age of 60 or more than 60 years old	The body weight gain more than 2kg a year: Yes	0.026963
The plasma ALT levels no greater than 40IU/L	The use of rosuvastatin	The urinary protein:Borderline	0.027764
The exercise habits more than 30min per day: No	The age of 60 or more than 60 years old	The plasma triglyceride levels less than 150 mg/dL	0.028303
The body mass index between 25.0-30.0	The gender: Male	The use of aspirin	0.029303

The plasma ALT levels no greater than 40IU/L	The plasma LDL cholesterol levels less than 140 mg/dL	The use of rosuvastatin	0.029591
The plasma AST levels no greater than 40IU/L	The use of aspirin	The plasma triglyceride levels between 150-300mg/dL	0.029799
The gender: Male	The use of aspirin	The plasma triglyceride levels between 150-300mg/dL	0.029799
The plasma HDL cholesterol levels more than 40mg/L	The use of rosuvastatin	The plasma γ-GTP levels no greater than 71IU/L	0.029855
The drinking alcohol at present: None	The age of 60 or more than 60 years old	The urinary protein:Borderline	0.030806
The habitual smoking: No	The systolic blood pressure between 90-110mmHg	The urinary glucose: Borderline levels	0.030875
The urinary glucose: Borderline	The systolic blood pressure between 90-110mmHg	The plasma triglyceride levels less than 150 mg/dL	0.030953
The use of aspirin	The plasma triglyceride levels less than 150 mg/dL,	The urinary protein:Borderline	0.031187
The body weight gain more than 2kg a year: No	The plasma ALT levels no greater than 40IU/L	The gender: Male	0.031583
The body mass index between 25.0-30.0	The age of 60 or more than 60 years old	The plasma γ-GTP levels no greater than 71IU/L	0.031934
The gender: Male	The exercise habits more than 30min per day: No	The urinary glucose: Borderline levels	0.032587
The gender: Male	The drinking alcohol at present: None	The urinary glucose: Borderline levels	0.032587
The chest XP: Normal	The use of aspirin	VDT: Need for the observation	0.03295
The body mass index between 25.0-30.0	The plasma AST levels no greater than 40IU/L	The use of aspirin	0.03295
The habitual smoking: No	The plasma HbA1c levels no greater than 6.2%	The use of warfarin	0.03295
The use of rosuvastatin	The use of warfarin	The plasma γ-GTP levels no greater than 71IU/L	0.033888
The gender: Male	The use of rosuvastatin	The use of warfarin	0.033888
The urinary protein:Borderline	The use of warfarin	The plasma γ-GTP levels no greater than 71IU/L	0.03457
The urinary glucose: Borderline	The use of rosuvastatin	The plasma γ-GTP levels no greater than 71IU/L	0.034591

The plasma AST levels no greater than 40IU/L	The use of rosuvastatin	The plasma γ-GTP levels no greater than 71IU/L	0.034591
The gender: Male	The plasma LDL cholesterol levels between 140-200mg/dL	The use of aspirin	0.034779
The age of 60 or more than 60 years old	The plasma triglyceride levels less than 150 mg/dL,	The body weight gain more than 2kg a year: Yes	0.035089
The body weight gain more than 2kg a year: No	The gender: Male	The fasting plasma glucose levels less than 110mg/dL	0.036414
The use of aspirin	VDT: Need for the observation	The urinary glucose: Borderline levels	0.036986
The plasma HbA1c levels no greater than 6.2%	The plasma AST levels no greater than 40IU/L	The use of warfarin	0.036986
The systolic blood pressure more than 140mmHg			0.037029
The gender: Male	The drinking alcohol at present: None	The age of 60 or more than 60 years old	0.037313
The plasma LDL cholesterol levels less than 140 mg/dL	The exercise habits more than 30min per day: No	The age of 60 or more than 60 years old	0.038212
The use of aspirin	VDT: Need for the observation	The plasma triglyceride levels between 150-300mg/dL	0.038984
The use of aspirin	The urinary glucose: Borderline	The plasma triglyceride levels between 150-300mg/dL	0.039984
The plasma triglyceride levels less than 150 mg/dL,	The use of warfarin	The urinary protein:Borderline	0.039984
The plasma LDL cholesterol levels less than 140 mg/dL	The diastolic blood pressure less than 90mmHg	The use of warfarin	0.039984
The gender: Male	The exercise habits more than 30min per day: No	The diastolic blood pressure between 90-110mmHg	0.04142
The habitual smoking: No	The plasma AST levels no greater than 40IU/L	The use of warfarin	0.041446
The age of 60 or more than 60 years old	The plasma HbA1c levels no greater than 6.2%	The use of aspirin	0.041962
The plasma ALT levels no greater than 40IU/L	The plasma LDL cholesterol levels between 140-200mg/dL	The use of aspirin	0.042269
The plasma LDL cholesterol levels less than 140 mg/dL	The age of 60 or more than 60 years old	The use of warfarin	0.042269
The chest XP: Normal	The use of warfarin	The plasma HDL cholesterol levels more than 40 mg/dL	0.042269

The plasma HDL cholesterol levels more than 40mg/L	VDT: Need for the observation	The use of warfarin	0.042269
The chest XP: Normal	VDT: Need for the observation	The use of warfarin	0.042269
The gender: Male	The exercise habits more than 30min per day: No	The age of 60 or more than 60 years old	0.042339
The body weight gain more than 2kg a year: No	The gender: Male	The drinking alcohol at present: Everyday	0.042769
The habitual smoking: No	The plasma LDL cholesterol levels less than 140 mg/dL	The use of aspirin	0.044975
The plasma LDL cholesterol levels less than 140 mg/dL	The plasma AST levels no greater than 40IU/L	The use of rosuvastatin	0.045047
The gender: Male	The drinking alcohol at present: None	The exercise habits more than 30min per day: No	0.045742
The plasma ALT levels no greater than 40IU/L	The age of 60 or more than 60 years old	The use of aspirin	0.046019
The use of rosuvastatin			0.046023
The gender: Male	The use of warfarin	The plasma γ-GTP levels no greater than 71IU/L	0.04611
The gender: Male	The use of aspirin	VDT: Need for the observation	0.046368
The body mass index between 25.0-30.0	The use of aspirin	The plasma HDL cholesterol levels more than 40 mg/dL	0.046368
The gender: Male	The drinking alcohol at present: None	The plasma γ-GTP levels no greater than 71IU/L	0.047455
The drinking alcohol at present: None	The age of 60 or more than 60 years old	The body weight gain more than 2kg a year: Yes	0.047742

Abbreviations: ALT : alanine aminotransferase, AST : aspartate aminotransferase, γGTP : γ-glutamyl transpeptidase, LDL: low density lipoprotein, HbA1c : hemoglobin A1c, VDT: visual display terminals
